# Dissecting the genetic basis of comorbid epilepsy phenotypes in neurodevelopmental disorders

**DOI:** 10.1186/s13073-019-0678-y

**Published:** 2019-10-25

**Authors:** Julie Chow, Matthew Jensen, Hajar Amini, Farhad Hormozdiari, Osnat Penn, Sagiv Shifman, Santhosh Girirajan, Fereydoun Hormozdiari

**Affiliations:** 1 UC Davis Genome Center, University of California, Davis, USA; 2 Bioinformatics and Genomics Graduate Program, The Huck Institutes of the Life Sciences, Pennsylvania State University, University Park, USA; 3 Department of Neurology, School of Medicine, University of California, Davis, USA; 4 T.H. Chan School of Public Health, Harvard University, Boston, USA; 5 MyHeritage, 6037606 Or Yehuda, Israel; 6 Department of Genetics, The Institute of Life Sciences, The Hebrew University of Jerusalem, Jerusalem, Israel; 7 Department of Biochemistry and Molecular Biology, Pennsylvania State University, University Park, USA; 8 MIND Institute, University of California, Davis, USA; 9 Biochemistry and Molecular Medicine, University of California, Davis, USA

**Keywords:** Epilepsy, Autism, Intellectual disability, Developmental disability, Module discovery, De novo mutation

## Abstract

**Background:**

Neurodevelopmental disorders (NDDs) such as autism spectrum disorder, intellectual disability, developmental disability, and epilepsy are characterized by abnormal brain development that may affect cognition, learning, behavior, and motor skills. High co-occurrence (comorbidity) of NDDs indicates a shared, underlying biological mechanism. The genetic heterogeneity and overlap observed in NDDs make it difficult to identify the genetic causes of specific clinical symptoms, such as seizures.

**Methods:**

We present a computational method, MAGI-S, to discover modules or groups of highly connected genes that together potentially perform a similar biological function. MAGI-S integrates protein-protein interaction and co-expression networks to form modules centered around the selection of a single “seed” gene, yielding modules consisting of genes that are highly co-expressed with the seed gene. We aim to dissect the epilepsy phenotype from a general NDD phenotype by providing MAGI-S with high confidence NDD seed genes with varying degrees of association with epilepsy, and we assess the enrichment of de novo mutation, NDD-associated genes, and relevant biological function of constructed modules.

**Results:**

The newly identified modules account for the increased rate of de novo non-synonymous mutations in autism, intellectual disability, developmental disability, and epilepsy, and enrichment of copy number variations (CNVs) in developmental disability. We also observed that modules seeded with genes strongly associated with epilepsy tend to have a higher association with epilepsy phenotypes than modules seeded at other neurodevelopmental disorder genes. Modules seeded with genes strongly associated with epilepsy (e.g., *SCN1A*, *GABRA1*, and *KCNB1*) are significantly associated with synaptic transmission, long-term potentiation, and calcium signaling pathways. On the other hand, modules found with seed genes that are not associated or weakly associated with epilepsy are mostly involved with RNA regulation and chromatin remodeling.

**Conclusions:**

In summary, our method identifies modules enriched with de novo non-synonymous mutations and can capture specific networks that underlie the epilepsy phenotype and display distinct enrichment in relevant biological processes. MAGI-S is available at https://github.com/jchow32/magi-s.

## Background

Phenotypic heterogeneity in neurodevelopmental disorders (NDDs) has been well documented and includes variability in the severity of symptoms, age of onset, and comorbidity of distinct clinical phenotypes in affected individuals [1]. For example, more than 30% of individuals with autism spectrum disorders are estimated to have epilepsy [2], and individuals with epilepsy have an increased comorbidity of autism and intellectual disability/developmental disability (ID/DD) compared with individuals without epilepsy [3, 4]. The comorbidity of nosologically distinct phenotypes is reflected in an overlap of causative genes and the involvement of similar molecular processes for these disorders [5, 6]. For example, *SCN2A*, the causative gene for epilepsy-associated Dravet syndrome, is also a primary candidate gene for familial autism [7, 8], while *NRXN1* has been associated with epilepsy as well as autism, schizophrenia, and developmental disability [9, 10]. In fact, nearly all genes with identified de novo mutations in epilepsy cases [11, 12] also have identified de novo mutations for other NDDs [13, 14].

While indicative of the shared biological pathways of NDDs, the high degree of pleiotropy for candidate NDD genes has made the classification of candidate genes and the discovery of novel genes towards distinct developmental features difficult. To date, several computational approaches have been devised to identify shared pathways of candidate genes for genetic disorders [15–23]. These approaches generally combine mutations identified from sequencing data of affected individuals with gene interaction networks and/or co-expression data to group genes with mutations in the same pathway. For example, we previously described MAGI, a tool to identify modules of genes significantly enriched for de novo variants in individuals with autism and ID/DD by integrating both protein-protein interaction networks and RNA sequencing data with variant calls [16]. Using this method, we identified distinct gene modules for signaling pathways and synaptic transmission from a set of de novo variants, and patients with mutations in these modules were observed to have more severe ID phenotypes than other patients. However, these methods do not allow for isolation of gene modules and pathways that are associated with a specific phenotype, such as epilepsy, compared with those that are more generally associated with multiple NDDs. Network and expression-based integration approaches that can accomplish this task are necessary to further understand the phenotypic heterogeneity of NDD-associated genes [1, 24].

Here, we present MAGI-S, an extension of our method MAGI, that identifies modules consisting of genes with high connectivity in the co-expression and protein-protein interaction networks that are also highly co-expressed with an input “seed gene.” We have used MAGI-S to predict potential NDD modules that might help in dissecting the wide phenotypic and genotypic heterogeneity of NDDs. Our approach is based on the assumption that variants in genes that are highly interacting in protein-protein interactions networks and are highly co-expressed during brain development have a higher chance of manifesting similar phenotypes than variants in genes with a low degree of interaction. Using diverse sets of known candidate NDD genes, we utilized MAGI-S to identify modules of genes that are associated with NDD and can dissect the epilepsy phenotypes in NDD. We found that (i) most modules are significantly enriched for de novo mutations in affected probands with NDDs versus unaffected siblings, (ii) the union of genes in all modules related to epilepsy contains novel gene candidates for epilepsy, and (iii) these modules can dissect the epilepsy phenotypes for some NDD cases. Based on this analysis, we provide evidence that studying modules of related genes can be useful for better understanding the biomolecular causes of epilepsy phenotypes in NDDs.

## Methods

### MAGI-S

We previously developed MAGI [16], a tool for predicting pathways and modules significantly enriched for de novo variants associated with NDDs in cases compared to controls [16]. MAGI is a randomized algorithm that constructs genetic modules containing a set of related genes that are highly co-expressed during brain development, highly connected in protein-protein interaction networks, have very few severe variants in control populations, and are significantly enriched among de novo variants in affected individuals. Specifically, MAGI first assigns a score *s*_*i*_ to each gene *i* based on the number of de novo variants present in the affected cases, while accounting for gene length and distribution of de novo non-synonymous mutation [16]. Next, MAGI finds a set of genes, *M*, that maximizes a standardized score of the selected genes ($\mathrm{i}.\mathrm{e}.,{S}_M=\frac{\sum_{i\in M}{s}_i}{\sqrt{\mid M\mid }}$) while satisfying the connectivity conditions for both protein interaction and co-expression networks.

**Fig. 1:**
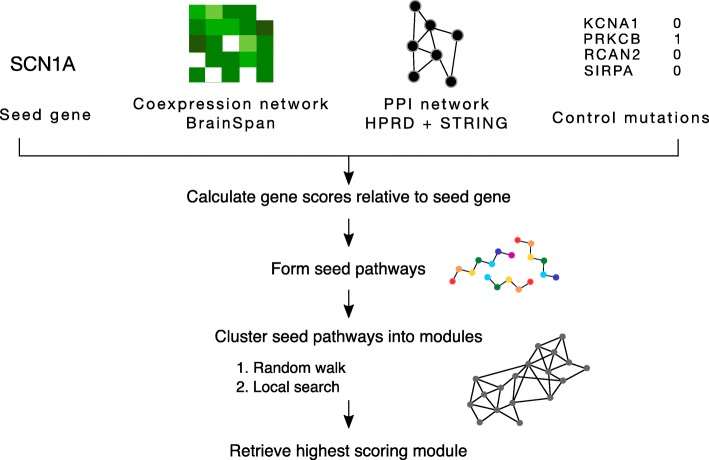
General overview of MAGI-S. A seed gene (e.g., SCN1A), protein-protein interaction (PPI) network, co-expression network, and LOF mutations in control samples are provided to MAGI-S to produce a seed centric module. Each gene in the PPI and co-expression networks is assigned a score based on the gene’s degree of co-expression with the seed gene relative to all other genes in the networks. Seed pathways are high-scoring simple paths formed from genes that are highly co-expressed relative to the seed gene, connected in the PPI network, and have a low number of LOF variants in control samples. Seed pathways are clustered into modules via a random walk of a graph created by seed pathways, and the total score of a module is improved by local search (similar to the MAGI algorithm in Hormozdiari et al. [16]). MAGI-S is run with varied parameters related to module size, minimum co-expression, and minimum PPI density, and the highest scoring module is retrieved. We have used the human developmental data from BrainSpan Atlas for the co-expression network construction. Furthermore, the combination of protein interactions from HPRD and STRING datasets was used as the PPI networks in our analysis

Here, we developed MAGI-S, a method which differs from MAGI in that MAGI-S uses a *known disease gene* as the input “seed gene” to identify a module that is highly co-expressed with the seed gene, rather than using de novo variants observed in affected cases, as in MAGI [16]. The objective of MAGI-S is to discover a set of genes (i.e., module) that share similar biological function with the *seed gene*. MAGI-S utilizes the co-expression network built using the BrainSpan Atlas of the Developing Human Brain [25], high-quality protein interactions from the Human Protein Reference Database and STRING, and loss-of-function (LOF) variants from a set of *normal*/*control* samples (see Additional file 1) (Fig. 1, Additional file 1: Figure S1) [26, 27].

MAGI-S assigns a score to each gene based on the relative ranking of the co-expression of that gene and the *input seed gene*. MAGI-S then finds a set of genes that are highly connected across interaction networks, have a low number of severe variants in control samples, and are highly co-expressed with the input seed gene. The MAGI-S algorithm, similar to MAGI, has two main steps. First, it finds a set of connected paths with a length between 5 and 8 genes in protein interaction networks that have a high summation of gene scores. Second, similar to MAGI, it utilizes a random walk and local search approach to cluster the constructed paths found in the first step into modules while satisfying the connectivity and co-expression constraints (see Additional file 1). This procedure is repeated, and the module with the highest score is selected.

#### Seed genes

MAGI-S allows any gene to be considered as the seed gene and produces modules centered around that gene. In this study, we consider over 100 well-known neurodevelopmental genes as input seed genes. We applied MAGI-S on a comprehensive set of seed genes known to contribute to NDDs found using different whole-exome and genome sequencing studies. We have considered all the genes reported in the SFARI gene list which were ranked as having the most evidence for contribution to autism by their analysis [28]. More formally, known NDD seed genes were selected from the following main databases: (i) the genes from SFARI Gene database with most evidence of contribution to NDD (i.e., gene scores of either 1 or 2 with a total of 84 genes), (ii) the genes that have been concurrently reported to be associated with epilepsy in OMIM, DDG2P, EpilepsyGene, and a recent review paper of epilepsy genes (total of 41 genes, 4 of which also have SFARI gene scores of either 1 or 2) [28–32], and (iii) an additional 6 genes moderately associated with epilepsy (*FLNA*, *FMR1*, *GRIN1*, *HNRNPU*, *NECAP1*, *NEDD1L*) (Additional file 1: Table S1). We have mainly focused on epilepsy as the phenotype of interest to investigate in patients with NDDs from discovered modules. In summary, we have considered a total of 127 genes which are known to be significantly associated with NDD phenotypes as input seed genes to MAGI-S. Due to a required minimum average co-expression, 16 potential seed genes failed to produce a module, yielding a total of 111 distinct modules*.* Note that many of these genes were selected based on the results available through whole-exome sequencing (WES) or whole-genome sequencing (WGS) of NDD cases/probands.

We first assigned the seed genes into three groups according to the known level of association with the epilepsy phenotype based on available disease-phenotype databases and literature [28, 30–34]. The three seed gene groups (classes) were defined based on reported epilepsy annotation from the following well-known sources: OMIM, DDG2P, EpilepsyGene, and Wang et al. [30, 31, 34]. We assigned the seed genes which were concurrently annotated by all four of these resources to be associated with epilepsy as *class 1*. Genes which were annotated to be associated in only a subset of the above resources were assigned to c*lass 2*. Finally, seed genes which were not associated with epilepsy in any of the above resources were assigned to *class 3* (Additional file 1: Table S1)*.*

These three different c*lasses* of seed genes represent the degree of evidence in the literature for their association with epilepsy phenotype. The specified grouping is based on the decreasing degree of known association with seizure of these seed genes as follows:

- *Class 1* (*ARHGEF9*, *ALDH7A1*, *ALG13*, *CACNA1H*, *CACNB4*, *CDKL5*, *CHD2*, *CHRNB2*, *DEPDC5*, *DNM1*, *EEF1A2*, *GABRA1*, *GABRB3*, *GABRG2*, *GNAO1*, *GRIN2A*, *GRIN2B*, *HCN1*, *KCNB1*, *KCNMA1*, *KCNQ2*, *KCNT1*, *KCTD7*, *LGI1*, *PCDH19*, *PRRT2*, *SCN1A*, *SCN1B*, *SCN2A*, *SCN8A*, *SLC25A22*, *SPTAN1*, *STX1B*, *STXBP1*, *TBC1D24*)
- *Class 2* (*ASH1L*, *BCKDK*, *CACNA1D*, *CNTNAP2*, *DIP2C*, *DYRK1A*, *FLNA*, *FMR1*, *GRIN1*, *HNRNPU*, *KMT2A*, *MBOAT7*, *MECP2*, *NECAP1*, *NEDD4L*, *PTEN*, *RANBP17*, *SCN9A*, *SLC6A1*, *SYNGAP1*, *TRIO*)
- *Class 3* (*ADNP*, *ANK2*, *ANKRD11*, *ARID1B*, *ASXL3*, *BAZ2B*, *BCL11A*, *CHD8*, *CIC*, *CTNND2*, *CUL3*, *DDX3X*, *DSCAM*, *ERBIN*, *GIGYF2*, *GRIA1*, *GRIP1*, *ILF2*, *INTS6*, *IRF2BPL*, *KDM5B*, *KDM6A*, *KMT2C*, *KMT5B*, *LEO1*, *MED13*, *MED13L*, *MET*, *MYT1L*, *NAA15*, *NCKAP1*, *NLGN3*, *NRXN1*, *PHF3*, *POGZ*, *RIMS1*, *SETD5*, *SHANK2*, *SHANK3*, *SMARCC2*, *SPAST*, *SRCAP*, *SRSF11*, *TAOK2*, *TBL1XR1*, *TBR1*, *TCF20*, *TNRC6B*, *TRIP12*, *UBN2*, *UPF3B*, *USP15*, *USP7*, *WAC*, *WDFY3*)

*Class 1* seed genes include genes which have the most indication of being involved with epilepsy-associated phenotypes based on available databases and literature [28, 30–34]. On the other hand, c*lass 3* are seed genes which have the least/no amount of evidence to be involved with the epilepsy phenotype based on the literature and are more associated with other neurodevelopmental phenotypes.

### Enrichment of de novo mutations and CNV from affected cases in modules

To assess the enrichment of de novo mutation in NDD cases relative to controls, de novo mutations were retrieved from denovo-db (version 1.6) [27]. denovo-db is a database of germline de novo variants that have been identified by next-generation sequencing technology aggregated from 54 different studies with rigorous phenotyping standards (Additional file 1). Variants within denovo-db have been curated to include information such as genomic position, reference and alternate alleles, functional category, associated phenotypes of the individual possessing the variant, and orthogonal validation status. The largest set of de novo variants used in our analysis are from the Simons Simplex Collection (SSC), which includes the de novo variation of both affected ASD probands and unaffected siblings. The other denovo-db studies used in our analysis have also had the highest quality of de novo call sets with a very low false discovery rate and similar rates of de novo variation. The complete set of missense (and missense-near-splice) or loss-of-function (frameshift, splice donor, splice acceptor, stop-gained, stop-gained-near-splice, stop-lost) mutations from the denovo-db resource for Simons Simplex Collection set [35–41], Autism Sequencing Consortium (ASC) [42], MSSNG [43, 44], Deciphering Developmental Disorders (DDD) [29], Epi4K [11], Helbig et al. [45], intellectual disability studies [46–49], and schizophrenia studies [50–54] were considered. In total, we study 12,199 NDD patients with ASD, ID, DD, or epilepsy and 1933 sibling/control individuals (Additional file 2: Table S2: “denovo-db”).

To determine the enrichment of copy number deletions and duplications within NDD cases relative to controls, we retrieve a copy number variant (CNV) morbidity map previously constructed from 29,085 children with developmental delay and 19,584 controls [60]. We assess the intersection of CNVs with genes within each module (Additional file 1).

### Dissection of epilepsy phenotype by enrichment of epilepsy genes within modules

To evaluate the enrichment of de novo non-synonymous variation specific to different cohorts of NDDs within each module, we use Fisher’s exact test to measure the enrichment of de novo variation in probands with either (1) ASD, ID, or DD, or (2) epilepsy relative to controls. Additionally, to quantify the enrichment of modules for genes with phenotypic annotations associated with either (1) NDDs without epilepsy or (2) NDDs with epilepsy, we calculated an enrichment score for each module as (*M*_*P*_/*M*_*P*′_)/(*G*_*P*_/(19, 986 − *G*_*P*_)), where *M*_*P*_ is the number of genes annotated as a certain NDD phenotype inside a module and *M*_*P*′_ is the complement, and *G*_*P*_ is the total number of genes annotated as a certain phenotype. There is a total of 19,986 protein-coding genes in the human genome (Gencode GRCh38.p12). Phenotypic annotations were retrieved from SFARI, OMIM, DDG2P, EpilepsyGene, or Wang et al. (Additional file 1) [28, 30–32].

### Pathway and ontology enrichment and expression analyses of modules

To describe pathway, gene ontology, and disease enrichment within a module, we provided a list of the genes within a module and its respective seed gene to Enrichr (http://amp.pharm.mssm.edu/Enrichr/) to produce pathway and GO biological process and Reactome pathway enrichments and OMIM disease annotations [55, 56]. We provided the same gene lists and the union of gene lists belonging to the same *class* to the cell type-specific expression analysis (CSEA), specific expression analyses (SEA), and tissue-specific expression analyses (TSEA) tools to assess the selective expression profiles of modules in the human brain and body [57].

## Results

We hypothesized that the phenotypic heterogeneity observed in NDDs can be better understood by dissecting the phenotype based on the pathways and modules disrupted in these disorders. Given the high comorbidity of NDDs, we tested the ability of MAGI-S to identify modules that can explain the association of specific genes to distinct phenotypes. Focusing on the more common comorbid feature of seizures, we applied MAGI-S to a subset of 111 seed genes strongly associated with NDDs, producing 1 module per seed gene. The size of the modules (i.e., the number of genes in each module) ranged from 25 to 79 genes, with an average size of 54 genes per module (Additional file 1: Figure S2).

### Significant enrichment of de novo mutations in neurodevelopmental modules

We used a set of well-known neurodevelopmental disorder genes as the input seed genes to MAGI-S for producing relevant modules [28, 30–32]. We then investigated if the identified modules as a whole were enriched with de novo mutations found in the largest independent NDD studies in denovo-db, including 8426 neurodevelopmental disorder patients from (1) Simons Simplex Collection (SSC), (2) MSSNG, and (3) Deciphering Developmental Disabilities (DDD) 2017 cohorts relative to 1933 sibling/control samples (data from denovo-db version 1.6, Additional file 2: Table S2: “denovo-db”) [27, 29, 43, 44, 58].

We compared the average number of loss-of-function (LOF), missense, and synonymous de novo mutations among probands and siblings/controls in the following sets: (1) the seed genes (total of 111 genes), (2) the union of all modules excluding seed genes (total of 1215 genes), (3) the union of all the genes in modules excluding the seed genes and 128 genes previously reported as significantly associated with ASD, ID, or DD [28, 34, 37, 42, 59] (Additional file 2: Table S2: “established NDD genes”) (a total of 1184 genes), and (4) all other genes possessing de novo mutations outside of the union of all constructed modules (total of 17,758 genes).

**Fig. 2:**
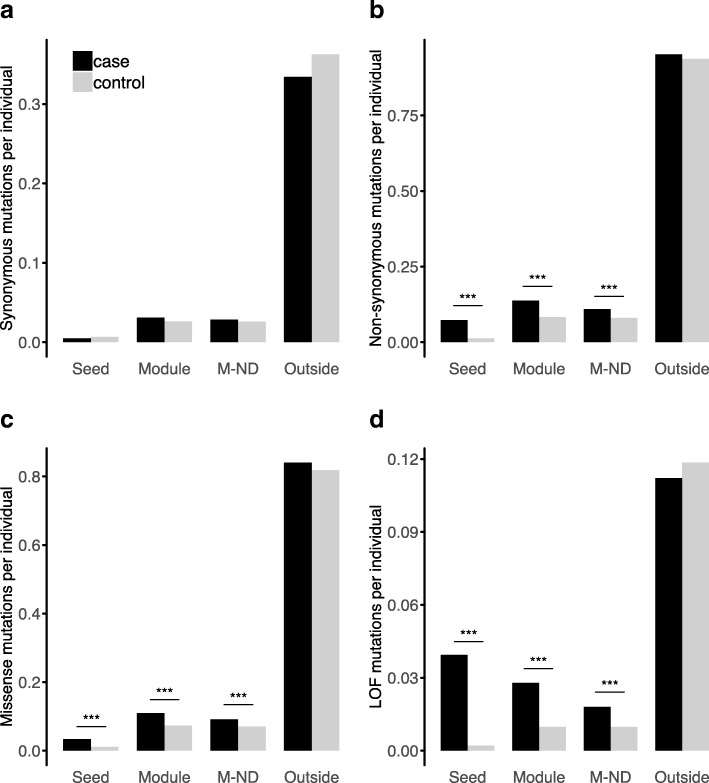
Average number of non-synonymous and synonymous de novo mutations per individual for probands and controls in seed genes (Seed), modules excluding seed genes (Module), module genes excluding 128 previously reported neurodevelopmental disorder genes (M-ND) (Additional file 2: Table S2: “established NDD genes”), and genes outside of any module (Outside). **a** No significant difference in the number of synonymous mutations exists between cases and controls. Cases display significantly more non-synonymous (**b**), including missense (**c**) and loss-of-function (**d**), variants than controls in the Seed, Module, and M-ND groups

First, as expected, we observed a significant enrichment of de novo variants in probands versus siblings for the seed genes (*p* < 9.72e−52, *p* < 2.90e−12, and *p* < 1.68e−57, for non-synonymous, missense, and LOF variants, respectively) (Fig. 2). Second, more importantly, significant enrichment was observed for de novo variants disrupting the genes within these modules while excluding the seed genes (*p* < 1.25e−10, *p* < 2.32e−6, and *p* < 1.74e−8, for non-synonymous, missense, and LOF variants, respectively). Third, we also observed a significant enrichment of de novo mutations disrupting the union of genes in modules after excluding the seed genes and genes recently reported in the literature to be significantly enriched with de novo variants in NDDs (*p <* 2.67e−4, *p* < 3.35e−3, and *p* < 5.22e−3, for non-synonymous, missense, and LOF variants, respectively). We note that this indicates the set of genes identified in these modules, even after removing the seed genes and the known neurodevelopmental genes, is still enriched for de novo variants in affected probands versus unaffected siblings/controls. Thus, we conclude that the set of genes in these modules should be enriched in novel NDD genes. Finally, for the remaining set of 17,758 genes, we did not observe any significant difference in de novo non-synonymous or synonymous variation between affected probands and unaffected siblings/controls (Fig. 2). Due to an unequal ratio of cases (8426) to controls (1933) sampled, we performed bootstrapping for 20,000 iterations per comparison to estimate the accuracy of the reported average number of mutations per individual (Additional file 1), finding the same pattern of increased enrichment of de novo non-synonymous variation in cases relative to controls. We found that seed genes contribute to the largest percentage of NDD diagnosis, followed by module genes, indicating that the modules capture a significant proportion of de novo mutations that affect NDDs even while excluding identified ASD/ID/DD genes (Additional file 2: Table S2: “enrichment (union)”).

We also compared the proportions of de novo mutations associated with autism spectrum disorder (ASD) [35–44], intellectual disability (ID) [46–49], developmental disability (DD) [29], epilepsy [11, 45], and schizophrenia (SCZ) [50–54] in genes inside and outside of *each of the 111 modules independently*. A total of 12,199 NDD probands and 1933 sibling/control samples were examined (Additional file 2: Table S2: “denovo-db”) [58]. For missense and LOF variants annotated with ASD, ID, DD, or epilepsy phenotypes, we evaluated the contingency tables and observed that we have an odds ratio significantly greater than 1 (with *p* < 0.05) for a large fraction of these modules, indicating enrichment of de novo mutations in neurodevelopmental probands versus controls in each of these modules (Additional file 2: Table S2). Resampling of contingency tables by 5000 iterations of permutation testing supports an increased enrichment of non-synonymous de novo mutation in individual modules (Additional file 2: Table S2: “contingency permutation”).

### Dissection of epilepsy phenotype in neurodevelopmental disorders using genetic modules

**Fig. 3:**
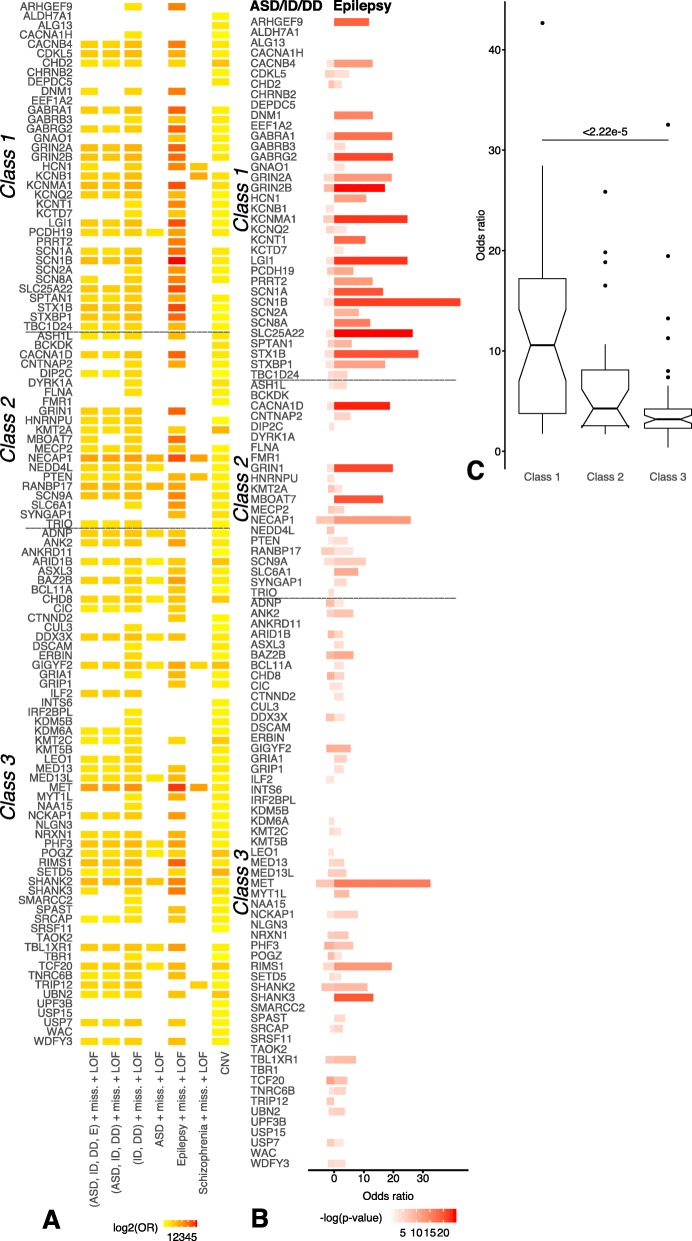
Summary of significant enrichment in de novo mutation and copy number variation (CNV) overlap in neurodevelopmental modules. Modules are grouped by class to indicate the degree of association of the seed gene with the epilepsy phenotype. Class 1, class 2, and class 3 modules correspond to the seed genes that have strong, moderate, and weak evidence of association with epilepsy, respectively. **a** Significant enrichment of missense (miss.) and loss-of-function (LOF) mutations for autism spectrum (ASD), intellectual disability (ID), developmental disability (DD), epilepsy (E), and schizophrenia cohorts within modules. **b** Comparison of log2 of significant (*p* < 0.05) enrichment of de novo mutation for variants annotated as ASD/ID/DD (left) or epilepsy (right). **c** Average odds ratio of de novo mutations annotated in epilepsy cases relative to controls is significantly greater in class 1 modules compared to class 3 modules

We next investigated the contribution of genetic modules in dissecting the epilepsy phenotype of NDDs. To assess the relevance of each of these 111 modules, we first measured the enrichment of de novo variants for ASD, ID, DD, epilepsy, and SCZ cohorts disrupting the modules selected for each of the seed genes (Fig. 3a). When considering any type of non-synonymous de novo variant associated with ASD, ID, DD, or epilepsy, we find that 64 of the 111 modules show significant enrichment in de novo non-synonymous mutations in these affected probands with neurodevelopmental disorders relative to unaffected siblings/controls (Fig. 3a). This shows that most of these modules (64/111 > 61%) are indeed significantly enriched in de novo mutations observed in the neurodevelopmental disorder probands versus unaffected siblings/controls. Furthermore, we observed that almost all modules (100/111 > 90%) are enriched in coding copy number variations (CNVs) that were detected via array comparative genomic hybridization (aCGH) in probands with developmental disorders relative to controls [60] (Additional file 2: Table S2). Additionally, probands with de novo non-synonymous mutations display (1) significantly lower verbal, non-verbal, or full-scale IQ in 30 of 111 modules and (2) an enrichment in macrocephaly in 7 of 111 modules relative to probands with de novo non-synonymous mutations outside of the modules (Additional file 2: Table S2). As expected, none of the modules are significantly enriched in synonymous mutations in probands relative to siblings/controls (Additional file 3: Table S3). In addition, enrichment of de novo non-synonymous mutation in cases relative to controls for each module was assessed for penetrant missense mutations with CADD score greater than 15 (Additional file 4: Table S2a), revealing increased de novo mutation burden in cases relative to controls in the union of all modules and in 64/111 individual modules (Additional file 1: Figure S3S-4).

We next studied the capacity of these modules to dissect the epilepsy phenotypes in neurodevelopmental disorders. We investigated the enrichment of non-synonymous de novo mutation in probands with either ASD/ID/DD or epilepsy (E) phenotype relative to controls. We first compared the odds ratio of de novo variants in ASD/ID/DD cohorts for each module to the odds ratio of de novo variants from the epilepsy cohort (Fig. 3). Note that *class 1* modules were constructed using neurodevelopmental seed genes with high evidence of association to epilepsy based on OMIM, DDG2P, and EpilepsyGene databases [28–32], whereas *class 3* modules were constructed using neurodevelopmental seed genes with minimal evidence of association with epilepsy in these databases. We compared the odds ratio of de novo variants observed in probands versus controls separately for the (1) ASD/ID/DD cohort and (2) the epilepsy cohort for each of the modules (Fig. 3b). The odds ratio for the ASD/ID/DD cohort is significantly greater than expected (*p* < 0.05) for 62 of 111 modules from all three classes. Similar fraction of modules from *classes 1*, *2*, and *3* had a higher than expected odds ratio for de novo mutations in the ASD/ID/DD cohort (19/35 > 54%, 13/21 > 61%, 30/55 > 54%, respectively).

On the other hand, a much larger fraction of modules from *class 1* (31/35 > 89%) had significantly greater than expected odds ratio for the de novo mutations in the *epilepsy cohort* (Fig. 3b). In contrast, the fraction of modules significantly enriched for de novo mutations is almost the same between ASD/ID/DDD and epilepsy cohorts for *class 3* modules (Fig. 3b).

We also compared the average odds ratio of modules for de novo non-synonymous variants in probands from the epilepsy cohort versus siblings/controls for modules in *class 1*, *class 2*, and *class 3* (Fig. 3c)*.* We observed a significantly higher average odds ratio for de novo variants in the epilepsy cohort for modules in *class 1* compared with *class 3* (*p* < 2.22e−5). These results support the hypothesis that modules predicted using seed genes can help in dissecting the epilepsy phenotype in neurodevelopmental disorders (Fig. 3b, c)*.*

#### Epilepsy genes enriched in modules built using *class 1* seed genes

**Fig. 4:**
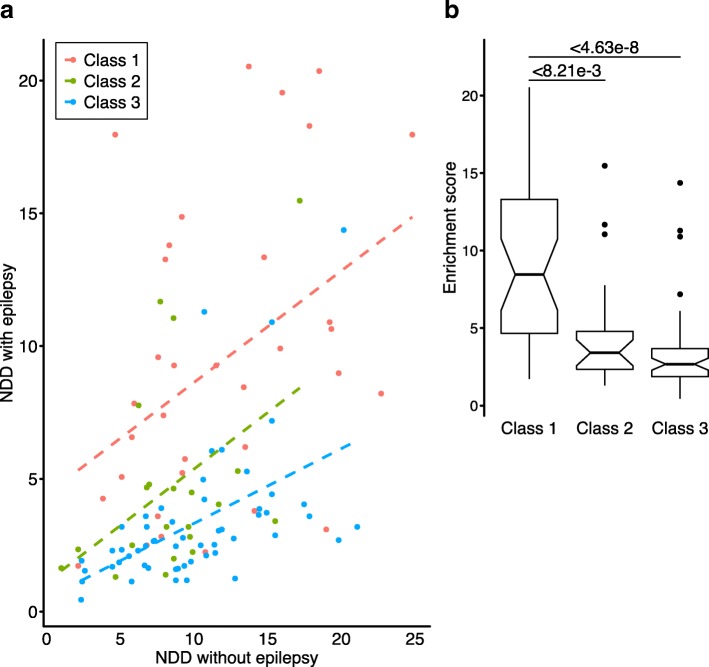
Phenotypic enrichment of genes in modules while including the seed gene. Enrichment is defined (*M*_*P*_/*M*_*P*′_)/(*G*_*P*_/(19,986 − *G*_*P*_)), where *M*_*P*_ is the number of genes annotated as a certain neurodevelopmental disorder (NDD) phenotype inside a module, *M*_*P*′_ is the complement of *M*_*P*_, and *G*_*P*_ is the total number of genes annotated as a certain phenotype. The total number of genes in the human genome is 19,986 (Gencode GRCh38.p12). Increased enrichment of NDD with or without epilepsy for a module corresponds respectively to the presence or absence of epilepsy phenotypes associated with the seed gene. Modules are grouped by evidence of epilepsy association of the seed genes—i.e., class 1 (strong), class 2 (moderate), and class 3 (weak association). **a** Increased enrichment of NDDs with epilepsy observed in class 1 modules are indicated by an increased *y*-intercept of class 1 regression line relative to class 2 and class 3. **b** Average enrichment of NDD with epilepsy is significantly greater in class 1 modules compared to class 2 or class 3 modules

To investigate the ability of modules to dissect epilepsy phenotypes, we assessed the enrichment of the epilepsy genes in the modules predicted by MAGI-S. Modules seeded with genes from *class 1* gene set contain a significantly higher number of genes previously reported to be associated with epilepsy (Additional file 1) than modules found using seed genes from either *class 2* or *class 3* gene sets (Fig. 4) [28, 30, 32, 33]. Similarly, the average number of genes associated with epilepsy in *class 1* modules was significantly higher than that in *class 2* or *class 3* modules *(p* < 8.21e−3 and *p* < 4.63e−8, respectively). Genes most frequently shared among *class 1* modules (such as *DLG4*, *GRIN2A*, *PRKCB*, and *SNAP25*) have been associated with epilepsy, synaptic function, and neuronal processes [61–64].

### Modules show enrichment in neuronal and epileptic processes

To assess the biological relevance of the identified modules, we analyzed the Gene Ontology (GO) and Reactome pathway enrichment for genes in each of the modules. The study of genetic modules disrupted in NDDs enables the identification of biological processes and functions that most contribute to these disorders. Enrichments from *class 1* and *class 2* modules indicate processes relevant to epilepsy and seizures, including GABAergic, cholinergic, dopaminergic, glycinergic, noradrenergic, and serotonergic synaptic transmission, and postsynaptic, excitatory, and inhibitory chemical synaptic transmission (Additional file 5: Table S4) [55].

Most modules (22/27 > 81%) that contained a large proportion of genes associated with epilepsy (enrichment score greater than 7.5) were enriched in chemical synaptic transmission pathways (Additional file 1: Figure S5). On the other hand, all remaining modules were enriched for genes related to chromatin regulation and or axon guidance.

Furthermore, many of *class 1* and *class 2* modules (33/56 > 58%) were enriched for interleukin signaling (*p* < 0.0001), which has been previously associated with epilepsy, and the MAPK, Ras, and VEGFR2 signaling pathways [65–70] (Additional file 1: Figure S6). Notch and TGF-beta signaling pathways were primarily enriched in *class 2* and *class 3* modules.

Using Enrichr analysis [56], we found that most (18/35 > 51%) *class 1* modules are enriched for genes significantly associated with the OMIM disease terms “epilepsy,” “seizures,” or “ataxia.” Conversely, the genes that occur most commonly in *class 3* modules (*UBC*, *EP300*, *SMAD2*, *CSNK2A1*, and *ABL1*) are associated with autism and/or intellectual disability [62, 71–73], and most (44/55 = 80%) *class 3* modules are enriched for genes associated with the term “autism” (Additional file 5: Table S4). We believe these results support the hypothesis that modules and networks can be utilized to dissect the phenotypic heterogeneity observed in NDDs.

### Selective expression of specific cell types and regulation in neurodevelopmental modules

**Fig. 5:**
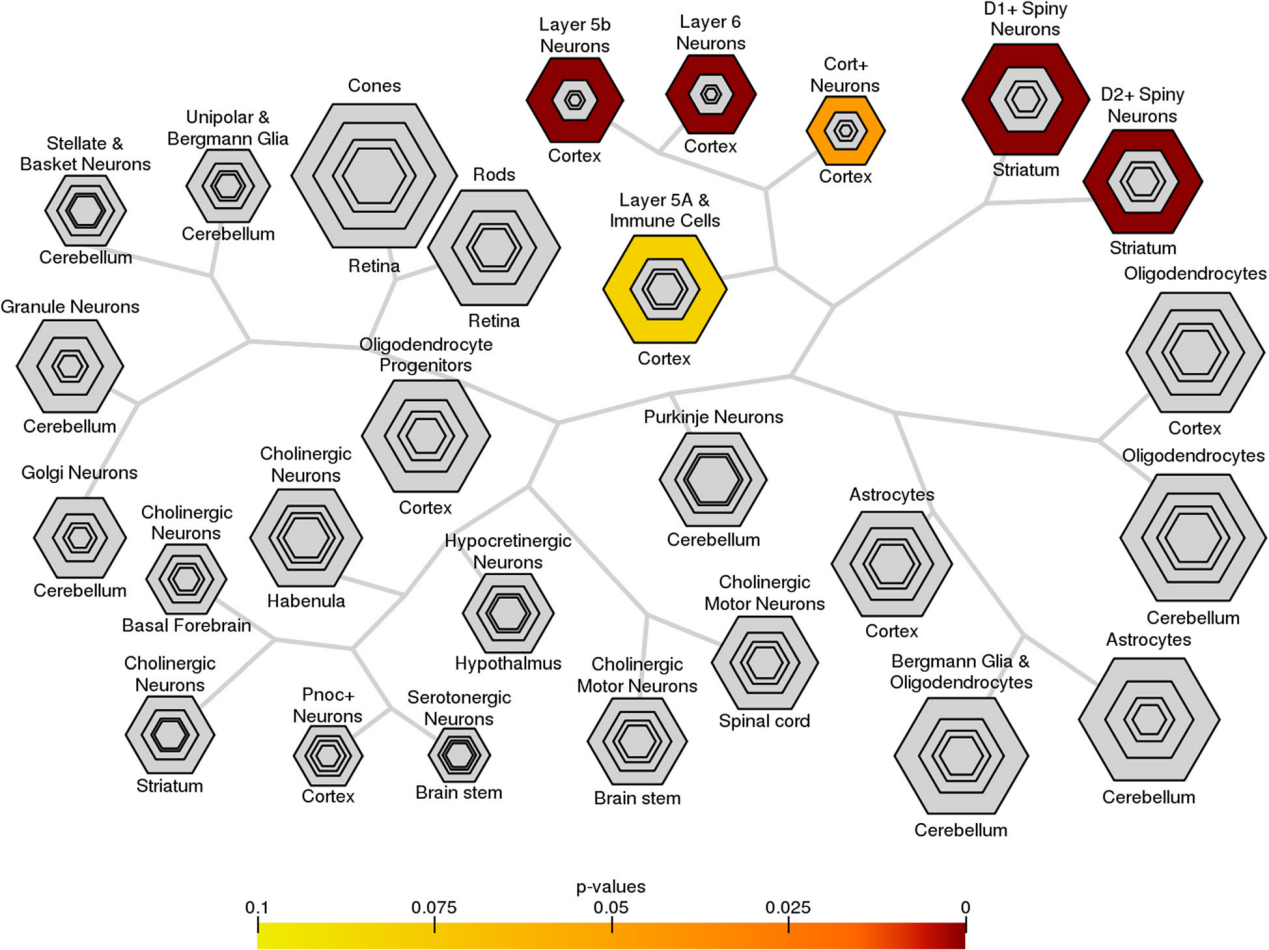
Cell type-specific expression analyses (CSEA) profile for the union of class 1 modules. Transcripts from provided gene lists that overlap significantly in specific cell types are indicated by intensity of color. Modules with seed genes strongly associated with epilepsy (class 1) show selective expression in the cortical neurons and spiny neurons in the striatum

We also sought to use our modules to pinpoint the neuronal critical cell types involved with specific neurodevelopmental disorder phenotypes. Knowing the neuronal cell types involved would help further study of gene expression dysregulation in those cell types in affected patients. We observed that most modules from *class 1* are selectively expressed in layer 5 and 6 cortical neurons and D1+ and D2+ spiny neurons in the striatum. This is also true for the union of the genes in all *class 1* modules (Fig. 5), according to the cell type-specific expression analyses (CSEA) tool that uses RNAseq data from BrainSpan [57]. Furthermore, genes in *class 1* modules show expression in early infancy to young adulthood in the developing brain, whereas genes in *class 2* and *class 3* modules separately are expressed primarily during fetal stages of development. Additionally, *class 1* modules are enriched specifically in the brain relative to other tissues, which complements the enrichment of pathways involved in growth and development in *class 3* modules (Additional file 1: Figure S7, Additional file 6: Table S5).

## Discussion

We have applied MAGI-S to construct modules from seed genes relevant to NDDs with and without association with epileptic phenotypes. The high degree of pleiotropy that exists among NDD genes complicates the understanding of the role of candidate genes in neurodevelopment. However, the ability to dissect the specific epilepsy phenotype from more general, heterogenous NDD phenotypes enables the improved characterization of candidate NDD genes in relation to specific NDD subtypes. To dissect specific phenotypes from a more general phenotype, the choice of seed genes with varied degrees of association with the specific phenotype of interest is critical to module discovery. MAGI-S produces modules that are highly co-expressed with the seed gene. Thus, we investigated the hypothesis that the selection of seed genes that are strongly associated with epilepsy would produce modules that participate in pathways that underlie the epilepsy phenotype. Seed genes were selected from the aggregation of different, large-scale studies, including whole-genome and whole-exome sequencing studies. It is important to note that the selected seed genes have a significant impact on the modules found by MAGI-S. To construct modules in a spatio-temporal context, we chose to use the BrainSpan Atlas that describes the gene co-expression in the developing human brain at a range of life stages [25]. Critical processes that underlie typical neurodevelopment are performed by co-expressed genes that may be vulnerable to deleterious mutation and are thus relevant to NDDs [74, 75]. As co-expression resources for affected probands at varying developmental stages are developed, modules constructed by MAGI-S will be able to more accurately reflect pathway dysregulation over time.

Genes that occur frequently and exclusively in *class 1* or *class 2* module groups point to potential novel candidate genes, such as *DLG4*, *PRKCB*, *STX1A*, and *YWHAH*, that may play important roles in NDDs with epileptic phenotypes. Among the genes that have not previously been defined as epilepsy genes, *DLG4* is the most commonly shared gene among *class 1* modules and has been implicated in autism, intellectual disability, and synaptic function [76, 77]. *PRKCB* is a candidate gene for partial epilepsies and possibly involved in microRNA dysregulation in patients with mesial temporal lobe epilepsy [63, 78]. *STX1A* is a presynaptic gene to which its paralog *STXBP1* binds to regulate the SNARE complex, associated with epilepsy [79], and *STX1A* knockout mice experience reduced dense-core vesicle exocytosis and abnormal monoaminergic transmission [80]. *YWHAx* genes, including *YWHAH*, have been hypothesized to be involved in neurological disorders including familial partial epilepsy [81, 82]. Genes that occur frequently in *class 3* modules but are absent in *class 1* modules such as *MYC* and *SIRT1* are implicated in tumorigenesis and metabolism [83, 84]; *SIRT1* is involved in learning and memory [85].

An accumulation of de novo missense and LOF mutations contribute to the manifestation of several NDDs [35, 86]. We found that most modules have significantly more de novo mutations in NDD probands than in controls (Fig. 2, Additional file 2: Table S2), and 88% of *class 1* modules are significantly enriched in epilepsy cohort-specific variants relative to controls. However, the potentially high degree of comorbidity among NDD probands and pleiotropy in NDDs suggests that particular de novo variants may impart the risk on several NDD phenotypes, although full comorbid phenotype information is not available from denovo-db. Thus, the enrichment of cohort-specific variants may not capture all genetic variation associated with a specific NDD phenotype, such as epilepsy. Analyses which concern all NDD-associated variants, such as the enrichment of de novo mutation within modules, are not dependent on phenotypic annotation and reflect the diversity of NDD phenotypes that may associate with seed genes and module genes. Seed genes and the union of all modules excluding seed genes capture a large proportion (~ 46%) of the de novo mutation signal that contributes to NDDs (Fig. 2, Additional file 2: Table S2: “enrichment (union)”). We observed that the union of genes identified in the modules is significantly enriched in de novo variants in NDD probands versus siblings/controls. This enrichment was still true after removing genes that were previously reported to be significantly enriched in de novo variants in these disorders (Additional file 2: Table S2: “established NDD genes”). However, we did not observe a significant difference between the genes not found in any module (17,758 genes) for de novo variants in NDDs versus siblings/controls. We believe this supports a polygenic model for de novo variation in NDDs, in which mutations accumulate in genes that modulate pathways that underlie complex disease, in comparison with an omnigenic model, in which disease-associated signals are widespread across the genome. The penetrance of rare genetic variation may also be affected by common variation to result in a wide phenotypic heterogeneity among NDDs with typically monogenic forms [87]. Additionally, the overlap of coding CNVs with individual modules, confirmed via permutation tests, indicates that there is a significantly greater proportion of CNVs that overlap genes inside modules in probands than in controls, suggesting that copy number variation of genes within modules may also disrupt normal neurodevelopmental function.

We assessed the relevance of modules by comparing enrichments in biological processes, signaling pathways, and selective expression in the human brain during different developmental stages. Modules seeded with genes that are strongly associated with epilepsy tend to cluster more distinctly than other module groups in relation to GO biological processes and Reactome pathways [55] (Additional file 1: Figure S5, Additional file 1: Figure S6). Most modules seeded with epilepsy genes are strongly related to chemical synaptic transmission, while modules produced with seed genes associated with other NDDs without epileptic phenotypes are related to chromatin organization and regulation, suggesting that the biological processes of a module correspond to its respective seed gene. Indeed, genome-wide analyses have previously associated autism genes with chromatin regulation [42, 59, 88, 89]. *Class 1* and *class 2* modules that have NDD with epilepsy enrichment scores consistently greater than 7.5 are enriched in similar biological processes involving chemical synaptic transmission and are selectively expressed in deep cortical neurons and spiny neurons in the striatum, which may indicate a stronger role of certain *class 2* seed genes in epilepsy than previously suggested. The selective expression of *class 1* modules in layer 5 and 6 cortical neurons is consistent with the epilepsy phenotype. Loss of excitatory neurons and the initiation of epileptic discharge have been observed in deep cortical layers, including layers 5 and 6, in individuals with epilepsy [90–92]. Additionally, in the striatum, direct and indirect neural pathways respectively modulate motor function via dopamine receptors D1 and D2 [93–95].

## Conclusion

We have constructed modules of high connectivity relevant to NDDs. The choice of gene used for seed module construction is critical to module formation. To minimize bias in selecting seed genes, we selected all high confidence and strong candidate NDD genes curated from multiple, high-quality whole-exome and genome sequencing studies as per the SFARI Gene database and all genes that have been reported to be concurrently associated with epilepsy according to OMIM, DDG2P, EpilepsyGene, and a recent review [28, 30–32]. From our choices of seed genes, we describe three general classes of modules by the strength of evidence of epilepsy association. We find that the majority of modules are significantly enriched in de novo mutations, and modules constructed with seed genes that are strongly associated with epilepsy tend to be (1) significantly enriched in de novo mutation from individuals affected by epilepsy relative to unaffected controls, (2) enriched in epilepsy-associated genes, and (3) enriched for biological function relevant to seizure. Genes with de novo mutations that have not been traditionally associated with NDDs but are present in modules constructed from relevant seed genes could play an important role in disease. Furthermore, MAGI-S may be applied to dissect the genetic complexity of other diseases characterized by specific clinical features and identify candidate genes in diseases with strong de novo mutation components. The seed-centric approach to module discovery integrates interaction networks and identifies a core set of genes strongly associated with phenotypes attributed to the seed gene and supported by biological evidence.

## Supplementary information

**Additional file 1:** Supplementary methods, **Figure S1-S7, and Table S1.**

**Additional file 2:** **Table S2.** Summary of analyses performed per module, including determinations of enrichment of de novo mutation, overlap with coding copy number variations. Module membership and frequency of occurrence for all genes selected in any module are displayed in the ‘modules’ tab. Number of cases and controls for ASD, ID, DD, and epilepsy cohorts within denovo-db are displayed in the ‘denovo-db’ tab. Contingency tables for Fisher’s exact test were constructed to assess the enrichment of de novo mutation and copy number variations in modules. Contingency table permutation empirical *p-values* are displayed in the ‘contingency permutations’ tab. Percent contribution to neurodevelopmental disorder diagnoses and comparison of average number of mutations per individual are displayed in the ‘enrichment (union)’ tab. Tabs corresponding to a module name show the total number of de novo variants, associated phenotype, type of variant, and neurodevelopmental disorder-related descriptions per module.

**Additional file 3:** **Table S3**. Proportions of synonymous mutations in neurodevelopmental cases relative to controls. Tabs correspond to modules and respective total number of synonymous de novo variants.

**Additional file 4:** **Table S2a.** Similar to Additional file 2: Table S2, Additional file 4: Table S2a displays a summary of analyses performed per module while requiring a CADD score greater than 15 for missense variants.

**Additional file 5:** **Table S4.** Significant GO terms, KEGG, and Reactome pathway enrichments, and OMIM disease terms per module (*p-value* < 0.05).

**Additional file 6:** Contains Table S5. Selective expression profiles for union of modules based on strength of epilepsy association (*Classes 1*, *2*, and *3* as C1, C2, and C3, respectively), including: Cell-type specific Expression Analyses (CSEA), Specific Expression Analyses (SEA) for adult brain regions and development, and Tissue-Specific Expression Analyses (TSEA).

## Data Availability

denovo-db (version 1.6) is available at http://denovo-db.gs.washington.edu/denovo-db/. SFARI gene scores are available at https://gene.sfari.org/database/gene-scoring/. OMIM annotations are available at https://www.omim.org. DDG2P annotations are available at https://decipher.sanger.ac.uk/ddd#ddgenes. Enrichr is hosted at https://amp.pharm.mssm.edu/Enrichr/. CSEA, SEA, and TSEA tools are available at http://genetics.wustl.edu/jdlab/csea-tool-2/. MAGI source code, PPI network, co-expression hash tables and dataset, and control mutations are available at https://eichlerlab.gs.washington.edu/MAGI/. MAGI-S source code is available at https://github.com/jchow32/magi-s.

## Abbreviations

NDD: Neurodevelopmental disorder
ASD: Autism spectrum disorder
DD: Developmental disability/disorder
ID: Intellectual disability
SCZ: Schizophrenia
LOF: Loss-of-function
CNV: Copy number variation
SSC: Simons Simplex Collection
DDD: Deciphering Developmental Disorders
ASC: Autism Sequencing Consortium
CSEA: Cell-type specific expression analysis
SEA: Specific expression analysis
TSEA: Tissue-specific expression analysis
