# Supplementary material for: Dissecting the genetic basis of comorbid epilepsy phenotypes in neurodevelopmental disorders
Source: Genome Med. 2019 Oct 25;11:65. doi: 10.1186/s13073-019-0678-y (PMC6815046; doi:10.1186/s13073-019-0678-y)
Supplement: Supplementary file 1 — Additional file 1: Supplementary methods, Figure S1-S7, and Table S1. [file 13073_2019_678_MOESM1_ESM.pdf]

Supplementary Methods

Dissecting the genetic basis of comorbid epilepsy phenotypes in neurodevelopmental disorders

Julie Chow[1], Matthew Jensen[2], Hajar Amini[3], Farhad Hormozdiari[4], Osnat Penn, Sagiv Shifman[5], Santhosh Girirajan[2,6], Fereydoun Hormozdiari[1,7,8]

[1]UC-Davis Genome Center, University of California, Davis, USA

[2]Bioinformatics and Genomics Graduate Program, The Huck Institutes of the Life Sciences, Pennsylvania State University, University Park, USA

[3] Department of Neurology, School of Medicine, University of California, Davis, USA

[4]T.H. Chan School of Public Health, Harvard University, Boston, USA

[5] Department of Genetics, The Institute of Life Sciences, The Hebrew University of Jerusalem, Jerusalem, Israel

[6]Department of Biochemistry and Molecular Biology, Pennsylvania State University, University Park, USA

[7]MIND Institute, University of California, Davis, USA

[8]Biochemistry and Molecular Medicine, University of California, Davis, USA

A total of 111 seed genes (*ADNP*, *ALDH7A1*, *ALG13*, *ANK2*, *ANKRD11*, *ARHGEF9*, *ARID1B*, *ASH1L*, *ASXL3*, *BAZ2B*, *BCKDK*, *BCL11A*, *CACNA1D*, *CACNA1H*, *CACNB4*, *CDKL5*, *CHD2*, *CHD8*, *CHRNB2*, *CIC*, *CNTNAP2*, *CTNND2*, *CUL3*, *DDX3X*, *DEPDC5*, *DIP2C*, *DNM1*, *DSCAM*, *DYRK1A*, *EEF1A2*, *ERBIN*, *FLNA*, *FMR1*, *GABRA1*, *GABRB3*, *GABRG2*, *GIGYF2*, *GNAO1*, *GRIA1*, *GRIN1*, *GRIN2A*, *GRIN2B*, *GRIP1*, *HCN1*, *HNRNPU*, *ILF2*, *INTS6*, *IRF2BPL*, *KCNB1*, *KCNMA1*, *KCNQ2*, *KCNT1*, *KCTD7*, *KDM5B*, *KDM6A*, *KMT2A*, *KMT2C*, *KMT5B*, *LEO1*, *LGI1*, *MBOAT7*, *MECP2*, *MED13L*, *MED13*, *MET*, *MYT1L*, *NAA15*, *NCKAP1*, *NECAP1*, *NEDD4L*, *NLGN3*, *NRXN1*, *PCDH19*, *PHF3*, *POGZ*, *PRRT2*, *PTEN*, *RANBP17*, *RIMS1*, *SCN1A*, *SCN1B*, *SCN2A*, *SCN8A*, *SCN9A*, *SETD5*, *SHANK2*, *SHANK3*, *SLC25A22*, *SLC6A1*, *SMARCC2*, *SPAST*, *SPTAN1*, *SRCAP*, *SRSF11*, *STX1B*, *STXBP1*, *SYNGAP1*, *TAOK2*, *TBC1D24*, *TBL1XR1*, *TBR1*, *TCF20*, *TNRC6B*, *TRIO*, TRIP12, *UBN2*, *UPF3B*, *USP15*, *USP7*, *WAC*, *WDFY3*) associated with neurodevelopmental disorders (NDDs) including autism spectrum disorders (ASD), intellectual disability (ID), developmental disability (DD), or epilepsy were selected to produce modules via MAGI-S. Seed genes were selected from the following databases: (i) all genes from SFARI Gene database with gene scores of either 1 (high confidence ASD gene) or 2 (strong candidate gene for ASD) (total of 84 genes), (ii) the genes that have been **concurrently** reported to be associated with epilepsy in 1) OMIM, 2) DDG2P, 3) EpilepsyGene, and 4) a recent review paper of epilepsy genes (total of 41 genes, 4 of which also have SFARI gene scores of either 1 or 2) (1-5), (iii) and an additional 6 genes associated with NDDs.

Due to few protein-protein interactions (PPIs) or co-expression values (**Figure S1**) associated with certain gene names (*ERBIN*, *IRF2BPL*, *KMT2A*, *KMT2C*, *KMT5B*, *MBOAT7*, *NAA15, SRSF11*),

respective alternate gene names (*ERBB2IP*, *C14orf4*, *MLL*, *SUV420H1*, *MLL3*, *LENG4*, *NARG1*, *SFRS11*) were provided to MAGI-S for module discovery. Parameters related to minimum size (20-35), minimum average co-expression value (0.425-0.52), and minimum PPI density (0.085-0.14) of modules were tested through multiple trials to identify the optimal module producing the highest score. Potential seed genes *CACNA1A*, *CACNA2D3*, *CHRNA2*, *CHRNA4*, *CNTN4*, *DEAF1*, *FOXP1*, *KAT2B*, *KATNAL2*, *MAGEL2*, *MSNP1AS*, *PTCHD1*, *RELN*, *SLC1A2*, *SZT2*, *WWOX*, were omitted from enrichment analysis and failed to produce modules due to average co-expression values below the specified range for minimum average co-expression value. Modules ranged in size from 25 to 79 genes (**Figure S2**). Genes within modules were renamed according to approved gene symbols for enrichment analyses.

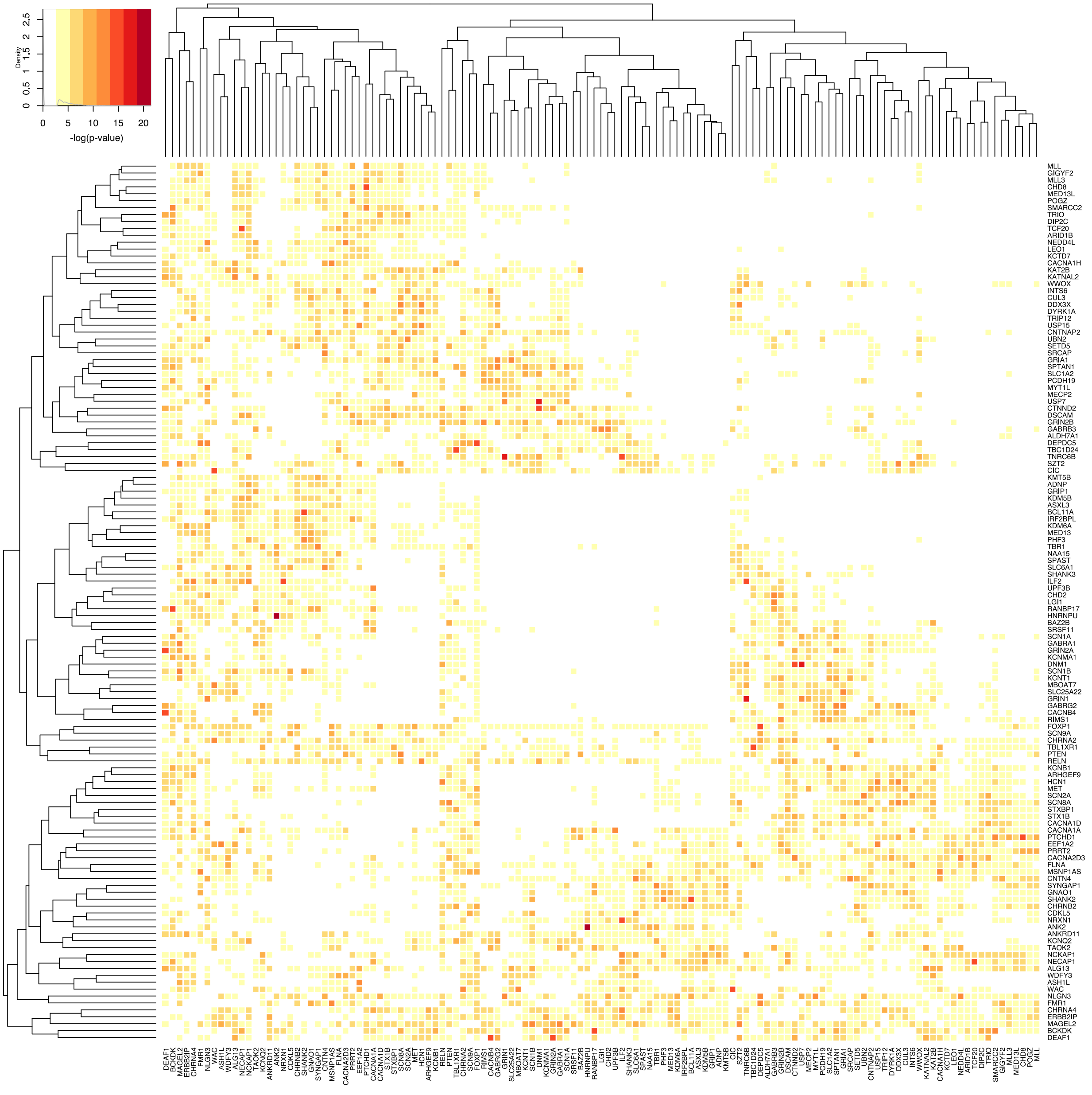

Figure S1. *Co-expression between seed genes. Co-expression values were determined by adjacency and Topological Overlap Matrix (TOM) matrices with power of 2 to reveal significant (p<0.05) co-expression among seed genes.*

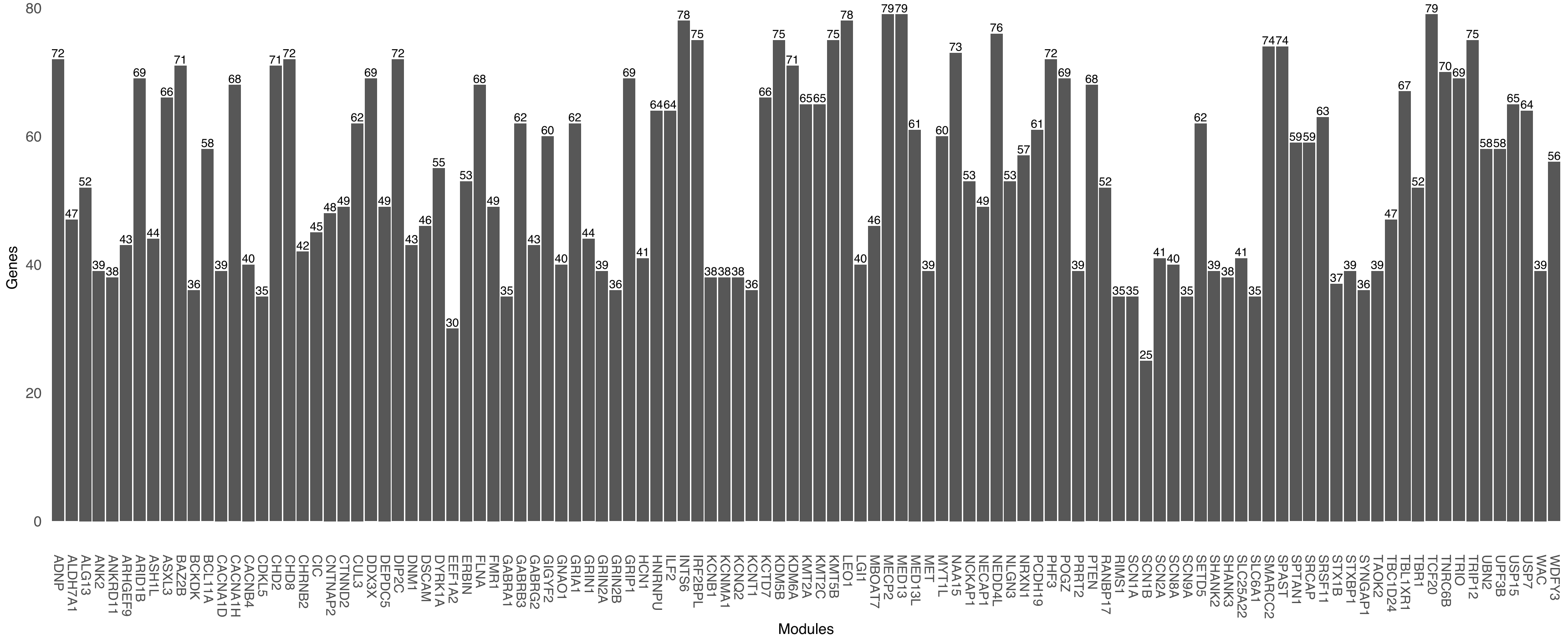

Figure S2. *Number of genes within each module excluding seed gene.*

Module groups (*Classes*) were defined by **concurrent** epilepsy annotations from the following sources (**Table S1**): *Class 1* (OMIM, DDG2P, EpilepsyGene, and Wang et al. 2017)*, Class 2* (a **subset** of *Class 1* sources), *Class 3* (**none** of *Class 1* sources) (3-5).

Determining enrichment of de novo mutations within modules

De novo mutations were retrieved from denovo-db (version 1.6) (6). The total number of missense (or missense-near-splice) or loss of function (frameshift, frameshift-near-splice, splice donor, splice acceptor, stop-gained, stop-gained-near-splice, stop-lost) mutations from the denovo-db Simons Simplex Collection (SSC) set (7-13), Autism Sequencing Consortium (ASC) (14), MSSNG (15, 16), Deciphering Developmental Disorders (DDD) (2), Epi4K (17), Helbig et al. 2016, and selected intellectual disability (18-21) and schizophrenia studies (22-26) were recorded.

Rigorous phenotyping standards were applied in contributing studies. The Autism Diagnostic Interview-Revised (ADI-R) and the Autism Diagnostic Observation Schedule (ADOS), among other measures (https://www.sfari.org/resources/ssc-instruments/), were recorded for probands with autism. For the SSC cohort, phenotyping was uniform across 12 university-affiliated clinics serving children with autism (27). For the Epi4K cohort, epilepsy phenotyping was accomplished by magnetic resonance imaging (MRI), electroencephalogram (EEG) findings, collection of medical records, and structured interviews (28). For intellectual disability cohorts, individuals with intellectual disability who were referred to a tertiary referral center for clinical genetics were further evaluated by a clinical geneticist (18), patients with intellectual disability were recruited by the Genetic Diagnostics Unit at Uppsala University Hospital (19), and patients with severe non-syndromic intellectual disability were selected from the German Mental Retardation

Network (20). For the developmental disability cohort, individuals with severe undiagnosed developmental disability were recruited, and phenotypes were described using the Human Phenotype Ontology (2). Patients in the schizophrenia cohort were recruited from psychiatric treatment settings (22-26).

We retrieved the total number of non-synonymous and synonymous mutations in genes in probands and controls and normalized the number of mutations by number of SSC, MSSNG, and DDD probands (8,426) and controls (1,933) considered (**Additional file 2: Table S2**: **'denovo-db'**). To compare the average number of *de novo* mutations per individual among probands and controls in 1) seed genes, 2) the union of all modules excluding seed genes, 3) the union of all modules excluding seed genes and 128 previously identified ASD/DD genes from the sources: de Rubeis et al. 2014, Mcrae et al. 2017 (DDD), O'Roak et al. 2014, Sanders et al. 2015, SFARI (score of 1) (1, 9, 14, 29, 30), and 4) outside of modules and seeds, we applied a one-tailed two-sample t-test on normalized counts of mutations per individual. To assess the accuracy of the t-test to measure true difference in normalized average number of mutations per individual, we applied 20,000 iterations of bootstrapping per comparison to calculate an empirical *p-value*. To determine an empirical *p-value*, we created bootstrap samples with replacement of cases (8,426) and controls (1,933) and calculated the t-test statistic for the bootstrapped sample and its respective *p-value*. If this *p-value* from the bootstrap sample was less than the *p-value* calculated prior to bootstrapping, then a 'total score' was incremented by one. The empirical *p*-value was then calculated as the total score divided by the number of iterations (20,000) plus 1.

We additionally constructed contingency tables of the raw counts of *de novo* mutations and evaluated Fisher's exact test to compare proportions of non-synonymous mutation among probands

and controls within the seed genes, the union of all modules excluding seed genes, and outside of modules. Percent contribution to the neurodevelopmental phenotypes was calculated by dividing the difference between the normalized number of mutations in probands and controls by the normalized number of mutations in probands (**Additional file Table S2: ‘enrichment (union)’**). We also assessed the average number of *de novo* mutation among probands and controls while requiring a CADD score greater than 15 for missense variants to examine likely penetrant non-synonymous mutations (**Figure S3**).

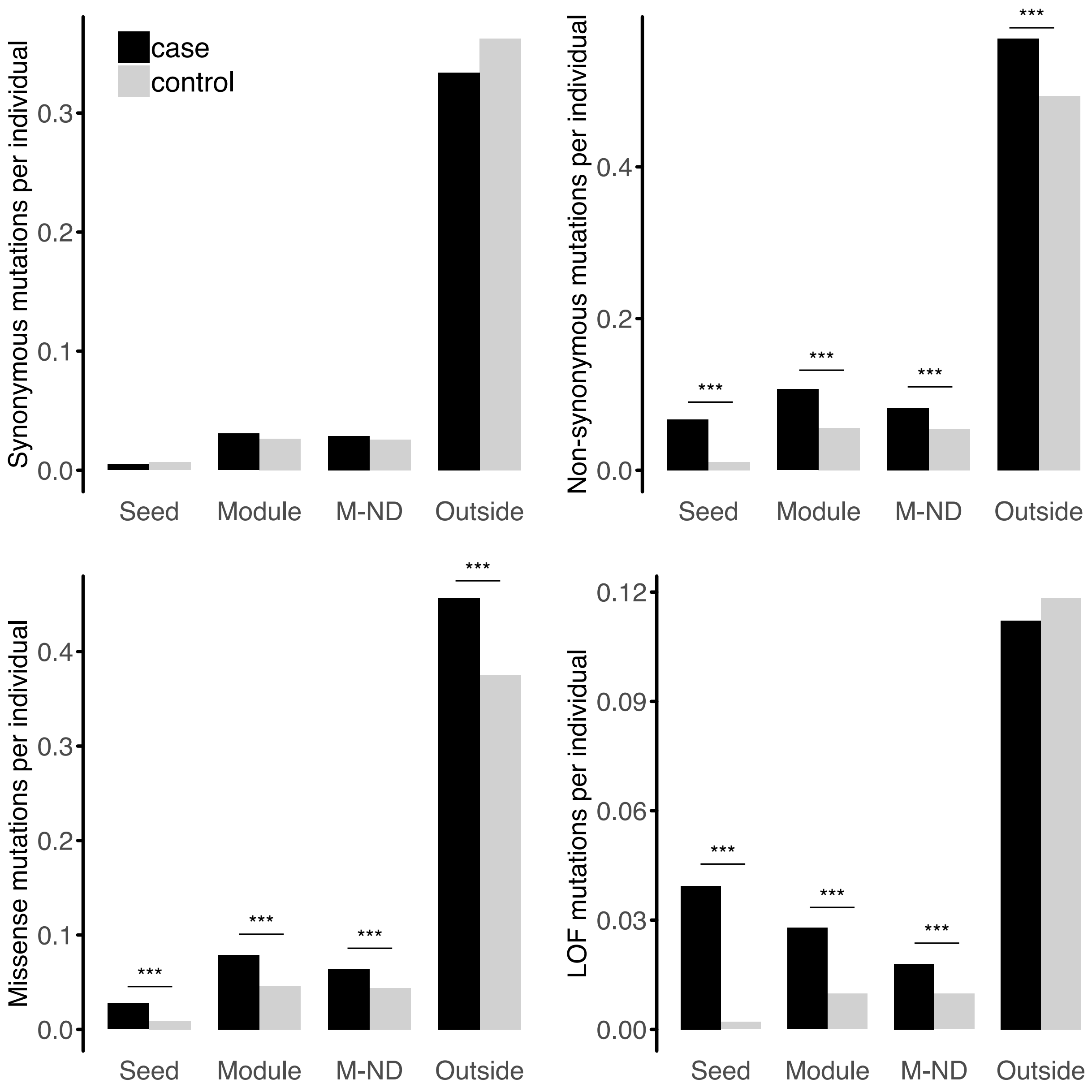

Figure S3. *Average number of non-synonymous and synonymous de novo mutations per individual for probands and controls in seed genes ('Seed'), modules excluding seed genes ('Module'), Module genes excluding 128 previously reported neurodevelopmental disorder genes ('M-ND'). Penetrant missense mutations are examined by requiring CADD score to be greater than 15.*

To determine if significant enrichment of non-synonymous *de novo* mutations within modules exists in probands with NDDs relative to controls, we compared the number of *de novo* missense and loss of function mutations inside and outside of the module via Fisher's exact test with consideration of a) only autism, developmental disorder, or intellectual disability variants (ASD, DD, ID), b) only ID or DD variants, c) only ASD variants, d) only epilepsy variants, and e) ASD, DD, ID and epilepsy variants (**Additional file 2: Table S2: 'denovo-db'**). Additionally, we further assessed the significance of *de novo* mutation enrichment in probands by considering a) missense or loss of function mutations, b) only missense, or c) only loss of function mutations. We repeated the above analyses while excluding variants attributed to the seed gene. To assess the accuracy of contingency tables applied to test the increased enrichment of non-synonymous mutation in cases relative to controls while excluding the seed gene, we applied resampling via 5,000 iterations of permutation testing per comparison. Cases and controls were randomly sampled indiscriminately to yield two sets of size equal to the number of cases and controls. Fisher's exact test was evaluated for each permuted set, and contingency tables were created to determine significant difference in proportions of non-synonymous mutation in or outside modules. We incremented a 'total score' for every permuted *p-value* less than the *p-value* calculated prior to permutation testing and calculated an empirical *p-value* as the total score divided by the number of iterations (5,000) plus 1.

The absence of any *de novo* mutations in controls in certain modules results in an infinitely large odds ratio. Thus, to better visualize significant enrichment of *de novo* mutation for modules with zero *de novo* mutations in controls, we increased the count of *de novo* mutation to one. We repeated the above analyses requiring missense variants to have a CADD score greater than 15 (**Figure S4**).

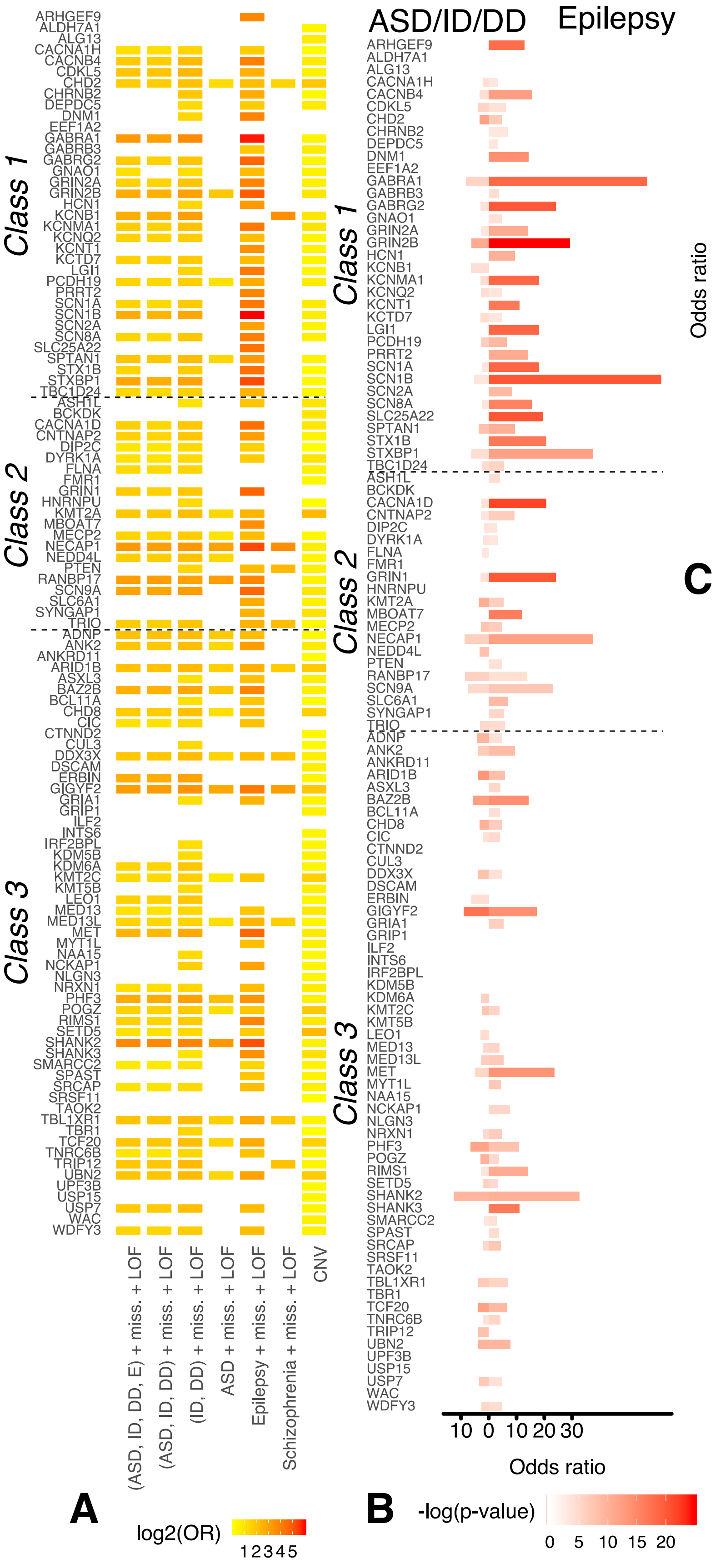

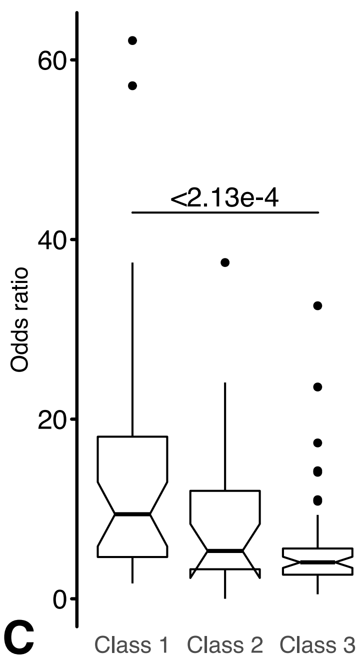

Figure S4. *Summary of significant enrichment in de novo mutation and copy number variation (CNV) overlap in neurodevelopmental modules for missense variants with CADD score greater than 15. Modules are grouped by Class to indicate degree of association of the seed gene with the epilepsy phenotype. Class 1, Class 2, and Class 3 modules correspond to seed genes that have strong, moderate, and weak evidence of association with epilepsy, respectively.* ***A)*** *Enrichment of missense (miss.) and loss of function (LOF) mutations for autism spectrum (ASD), intellectual disability (ID), developmental disability (DD), epilepsy (E), and schizophrenia cohorts within modules.* ***B)*** *Comparison of log2 of significant (p<0.05) enrichment of de novo mutation for variants annotated as ASD/ID/DD (left) or epilepsy (right).* ***C)*** *Average odds ratio of de novo mutations annotated in epilepsy cases relative to controls is significantly greater in Class 1 modules compared to Class 3 modules.*

Determining overlap of copy number variation morbidity map and modules

From a previously described copy number variant (CNV) morbidity map (31), we retrieve copy number deletions or duplications that overlap any of the genes within a module to determine if significant enrichment of coding copy number deletion and duplication exists in probands with developmental delay relative to copy number deletions in controls. We construct contingency tables to compare the proportion of coding CNVs in probands with CNVs from controls. To account for CNV burden in probands and controls, we conducted 5,000 permutation tests in which coding CNVs containing genes from the module of interest were randomly assigned to two groups of unequal size, with the size of each group corresponding to the number of coding CNVs in probands and in controls. Within a group, we determined how many CNVs contained genes inside or outside the module and constructed a contingency table. If the *p-value* of this contingency table was less than the initial observed *p-value*, then we increment a 'total score'. We calculate an empirical *p-value* by dividing the total score plus by the number of permutations plus 1. A significant empirical *p-value* indicates that an initial assessment of CNV enrichment as significant is indeed significant.

Assessing phenotypic differences in individuals with mutations within and outside modules

To determine if individuals with *de novo* missense or loss of function mutations within a module have lower IQ and higher Social Responsiveness Scale (SRS) T-scores than individuals with *de novo* mutations in genes outside of the module, we intersect Simons Simplex Collection (SSC) individuals with denovo-db and compare average verbal, non-verbal, and full scale IQ and SRS T-scores via a two-sample t-test (6, 27). To determine if the proportion of 1) male and female

individuals or 2) individuals with macrocephaly differs within a module, we conducted Fisher's exact tests for individuals with either missense or loss of function mutations and a phenotype of either autism, developmental disability, intellectual disability, or epilepsy. Macrocephaly scores were retrieved for SSC individuals, and scores > 3 were defined as macrocephalic.

Dissection of epilepsy phenotype by enrichment of epilepsy genes within modules

A gene was considered to have an epilepsy annotation if reported by OMIM or DDG2P to have an annotation of 'epilepsy', 'ataxia', 'seizure', or 'Ohtahara', or reported in EpilepsyGene or Wang et al. 2017 to be an epilepsy gene (3, 4). A gene was considered to have an ASD, ID, or DD annotation if the gene has a SFARI gene score of 1 or 2 (1), or is reported by OMIM or DDG2P (5) to be annotated with any of the following case-insensitive terms: autism, Angelman, fragile, intellect, Rett, retardation, Coffin, Bainbridge, CNOT3, Cognitive impairment, Cornelia, CSNK2A1, Developmental, Smith-Kingsmore, Feingold, Floating, GNAI1, Joubert, Kabuki, KBG, KCNQ3, KMT5B, Noonan, Megalencephaly-polymicrogyria-polydactyly-hydrocephalus, Mowat-Wilson, Myhre, Nijmegen, nonspecific severe ID, Opitz-Kaveggia, Phelan, Potocki-Shaffer, Riddle, Rubinstein, Temple-Barraister, Temple Barraister, Weaver, Wiedemann-Steiner, Woodhouse-Sakati, Tatton-Brown-Rahman, Aicardi-Goutieres, Au-Kline, CHOPS, CRASH, Dias-Logan, FG syndrome, Gabriele-de Vries, Helsmoortel-van der, Lopes-Maciel-Rodan, Kleefstra, Koolen-De Vries, Lujan-Fryns, Nicolaides-Baraitser, Pilarowski-Bjornsson, Pitt-Hopkins, Rubinstein-Taybi, Schuurs-Hoeijmakers, Seckel syndrome, Stankiewicz-Isidor, Takenouchi-Kosaki, White-Sutton, Witteveen-Kolk syndrome, You-Hoover-Fong.

Enrichment of NDDs with or without epilepsy was calculated by counting the number of genes within a module annotated with epilepsy or non-epilepsy associated terms with the formula $(M_P/M_{P'}) \ / \ (G_P \ / \ (19{,}986 - G_P))$, where M_P is the number of genes annotated as a certain NDD phenotype inside a module $M_{P'}$is the complement, and G_P is the total number of genes annotated as a certain phenotype. The total number of genes in the human genome (Gencode GRCh38.p12) is 19,986 genes.

As supplemental phenotypic descriptions, the terms 'epilepsy', 'seizure', 'ataxia', 'convulsion', 'autism', 'macrocephaly', 'intellectual', or 'neurodevelopment' were retained from the Mouse Genome Database (MGD), Mouse Genome Informatics, The Jackson Laboratory, Bar Harbor, Maine (http://www.informatics.jax.org/allele). MGD annotations were not considered in finding NDD phenotypic associations. SFARI gene scores ranging from minimal evidence (4) to high confidence (1) and DDG2P and OMIM descriptions are noted for genes within modules (1, 5).

Pathway and ontology enrichment and expression analyses of modules

Separate lists of genes within a module and respective seed genes were provided to Enrichr (http://amp.pharm.mssm.edu/Enrichr/) to produce pathway and GO biological process and Reactome pathway enrichments and OMIM disease annotations (**Figure S5, Figure S6**) (32, 33). Gene lists and the union of gene lists belonging to the same *Class* were provided to the Cell-type Specific Expression Analysis (CSEA), Specific Expression Analyses (SEA), and Tissue Specific Expression Analyses (TSEA) tools to assess selective expression profiles of modules in the human brain and body (**Figure S7**) (34). To visualize shared pathway and biological processes, we performed UPGMA hierarchical clustering on selected significant terms (p<0.0001) that occurred

in at least ten modules and were related to synapses, neurons, neurodevelopment, neurotransmitters, axons, chromatin, the brain, nervous system, potentiation, or signaling pathways.

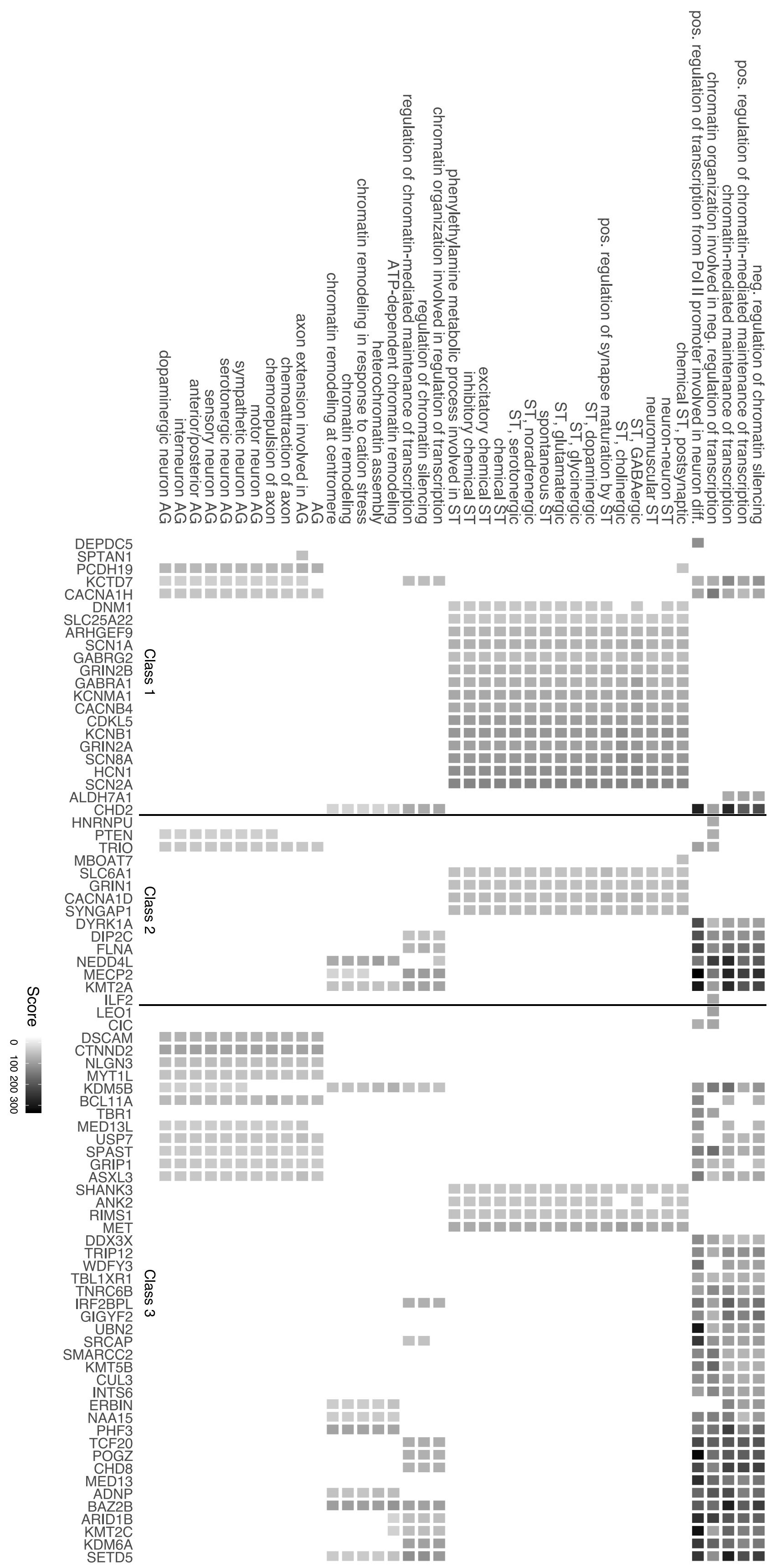

Figure S5. *Significant GO Biological Processes. GO terms related to neurodevelopment, synapses, and chromatin organization that are significantly enriched (p<0.0001) in at least 10 modules are displayed with combined enrichment scores calculated via Enrichr. Seed genes are grouped as Class 1, Class 2, and Class 3.*

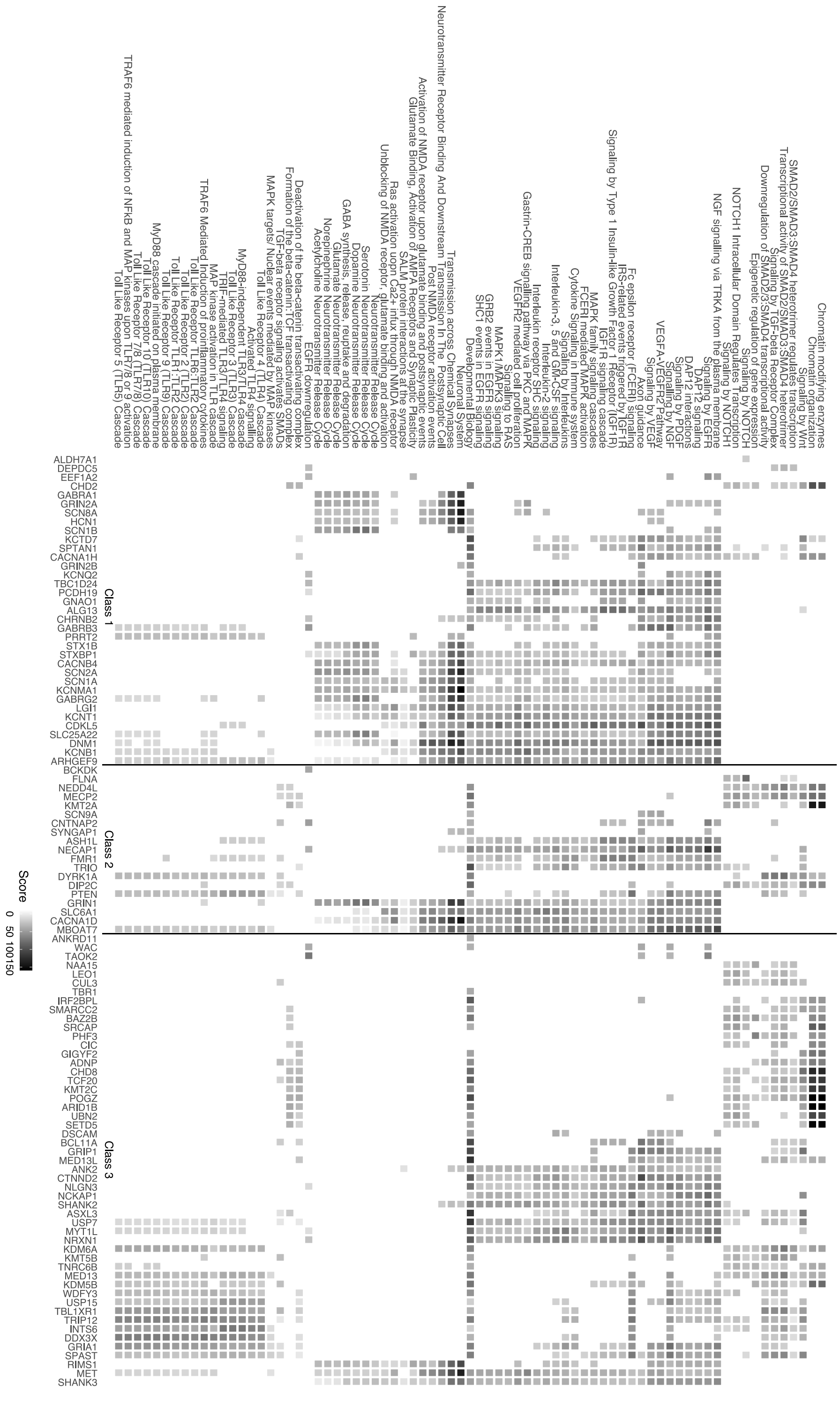

Figure S6. *Selected significant (p<0.0001) Reactome pathway terms present in at least 10 modules with combined Enrichr score.*

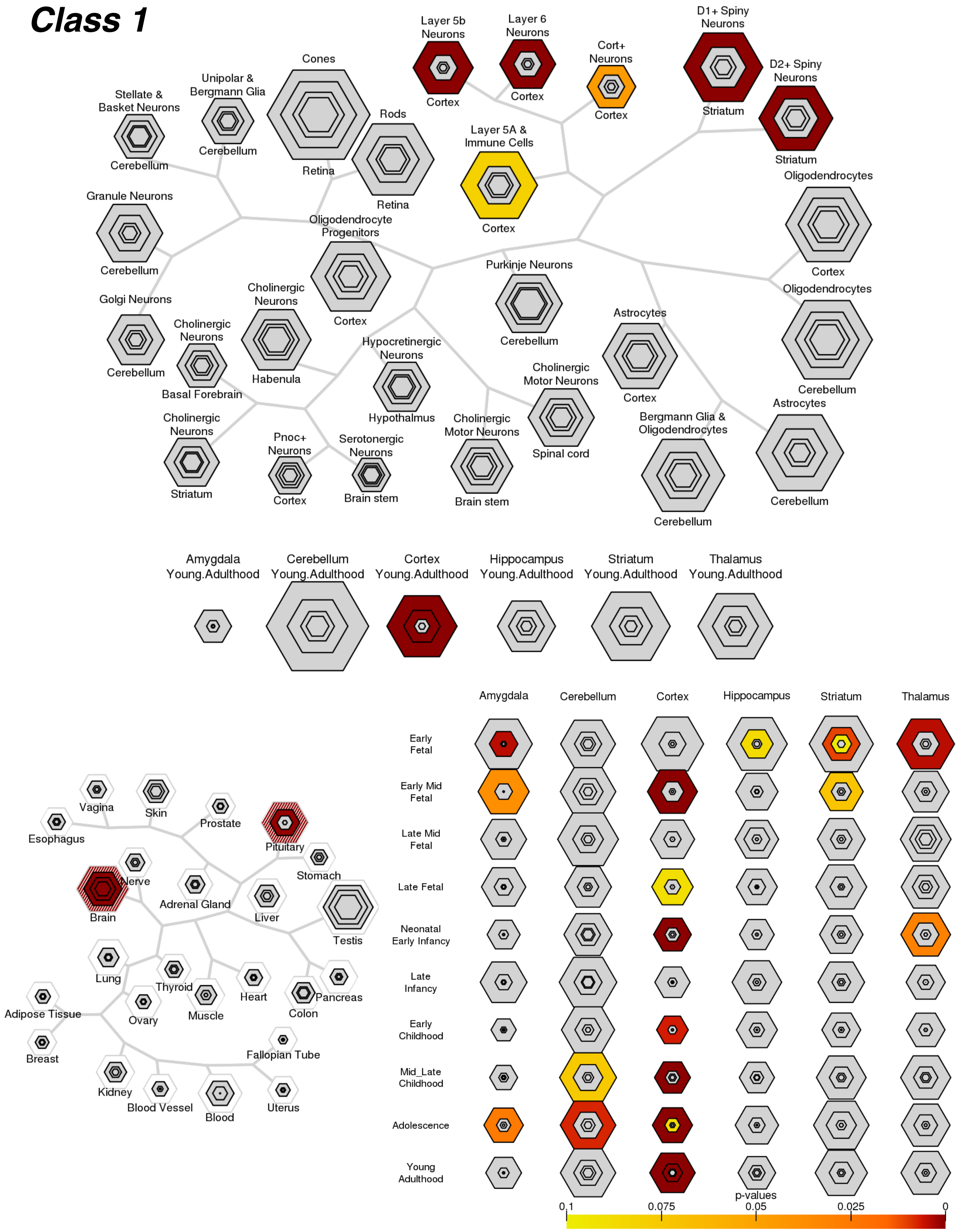

Class 1
Layer 5b Neurons
Cortex
Layer 6 Neurons
Cortex
Cort+ Neurons
Cortex
D1+ Spiny Neurons
Striatum
D2+ Spiny Neurons
Striatum
Stellate & Basket Neurons
Cerebellum
Unipolar & Bergmann Glia
Cerebellum
Cones
Retina
Rods
Retina
Layer 5A & Immune Cells
Cortex
Oligodendrocytes
Cortex
Granule Neurons
Cerebellum
Oligodendrocyte Progenitors
Cortex
Purkinje Neurons
Cerebellum
Oligodendrocytes
Cerebellum
Golgi Neurons
Cerebellum
Cholinergic Neurons
Habenula
Cholinergic Neurons
Basal Forebrain
Hypocretinergic Neurons
Hypothalmus
Astrocytes
Cortex
Cholinergic Motor Neurons
Spinal cord
Astrocytes
Cerebellum
Cholinergic Neurons
Striatum
Pnoc+ Neurons
Cortex
Serotonergic Neurons
Brain stem
Cholinergic Motor Neurons
Brain stem
Bergmann Glia & Oligodendrocytes
Cerebellum
Amygdala Young.Adulthood
Cerebellum Young.Adulthood
Cortex Young.Adulthood
Hippocampus Young.Adulthood
Striatum Young.Adulthood
Thalamus Young.Adulthood
Vagina
Skin
Esophagus
Prostate
Pituitary
Stomach
Nerve
Brain
Adrenal Gland
Liver
Testis
Lung
Thyroid
Muscle
Heart
Colon
Pancreas
Adipose Tissue
Ovary
Breast
Fallopian Tube
Kidney
Blood Vessel
Blood
Uterus
Amygdala
Cerebellum
Cortex
Hippocampus
Striatum
Thalamus
Early Fetal
Early Mid Fetal
Late Mid Fetal
Late Fetal
Neonatal Early Infancy
Late Infancy
Early Childhood
Mid_Late Childhood
Adolescence
Young Adulthood
p-values
0.1
0.075
0.05
0.025
0

Class 2

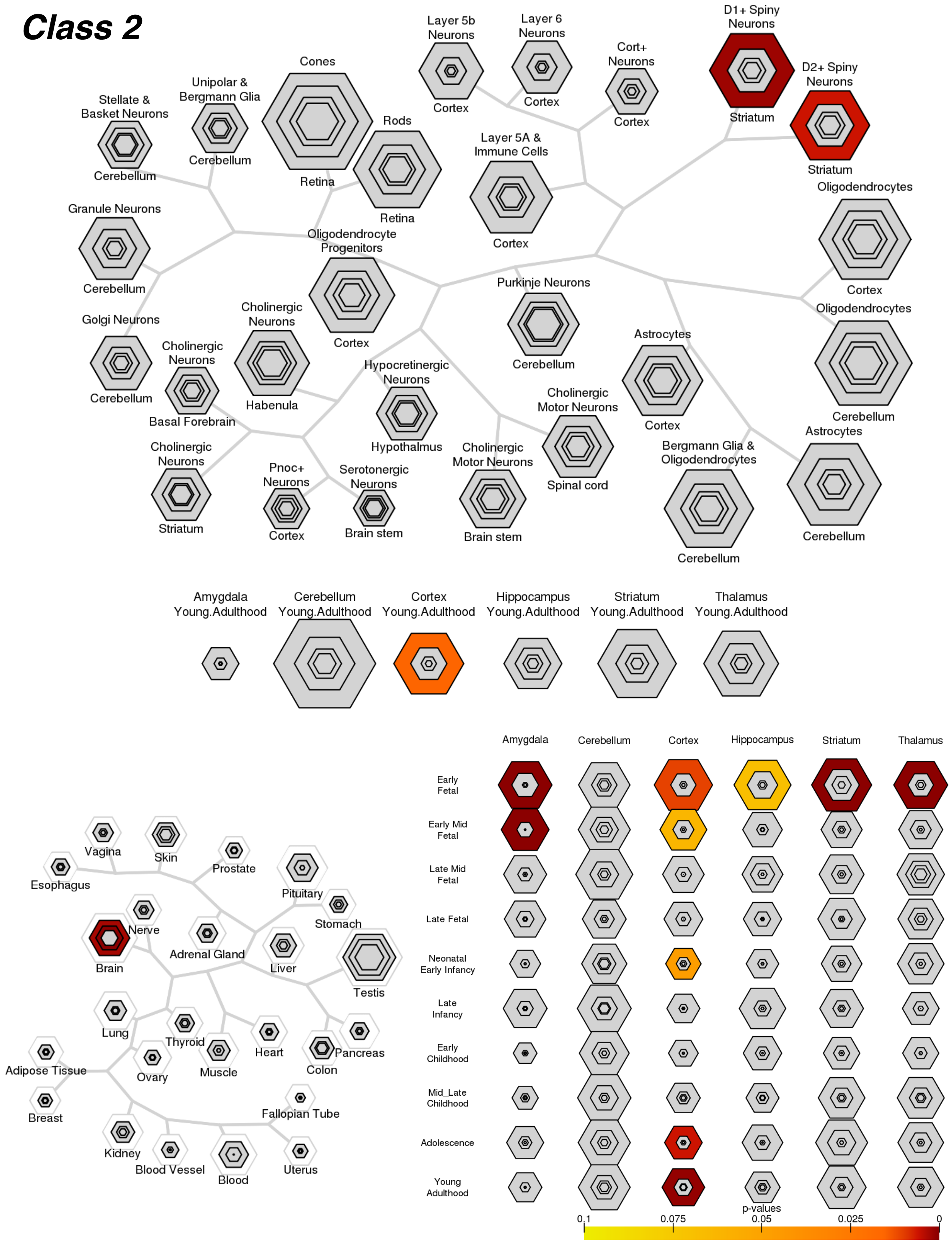

Stellate & Basket Neurons
Cerebellum
Unipolar & Bergmann Glia
Cerebellum
Cones
Retina
Rods
Retina
Layer 5b Neurons
Cortex
Layer 6 Neurons
Cortex
Cort+ Neurons
Cortex
D1+ Spiny Neurons
Striatum
D2+ Spiny Neurons
Striatum
Layer 5A & Immune Cells
Cortex
Granule Neurons
Cerebellum
Oligodendrocyte Progenitors
Cortex
Oligodendrocytes
Cortex
Oligodendrocytes
Cerebellum
Golgi Neurons
Cerebellum
Cholinergic Neurons
Habenula
Cholinergic Neurons
Basal Forebrain
Purkinje Neurons
Cerebellum
Astrocytes
Cortex
Astrocytes
Cerebellum
Hypocretinergic Neurons
Hypothalmus
Cholinergic Motor Neurons
Spinal cord
Cholinergic Motor Neurons
Brain stem
Bergmann Glia & Oligodendrocytes
Cerebellum
Cholinergic Neurons
Striatum
Pnoc+ Neurons
Cortex
Serotonergic Neurons
Brain stem
Amygdala Young.Adulthood
Cerebellum Young.Adulthood
Cortex Young.Adulthood
Hippocampus Young.Adulthood
Striatum Young.Adulthood
Thalamus Young.Adulthood
Vagina
Skin
Esophagus
Prostate
Pituitary
Stomach
Nerve
Brain
Adrenal Gland
Liver
Testis
Lung
Thyroid
Muscle
Heart
Colon
Pancreas
Adipose Tissue
Ovary
Breast
Fallopian Tube
Kidney
Blood Vessel
Blood
Uterus
Amygdala
Cerebellum
Cortex
Hippocampus
Striatum
Thalamus
Early Fetal
Early Mid Fetal
Late Mid Fetal
Late Fetal
Neonatal Early Infancy
Late Infancy
Early Childhood
Mid_Late Childhood
Adolescence
Young Adulthood
p-values
0.1
0.075
0.05
0.025
0

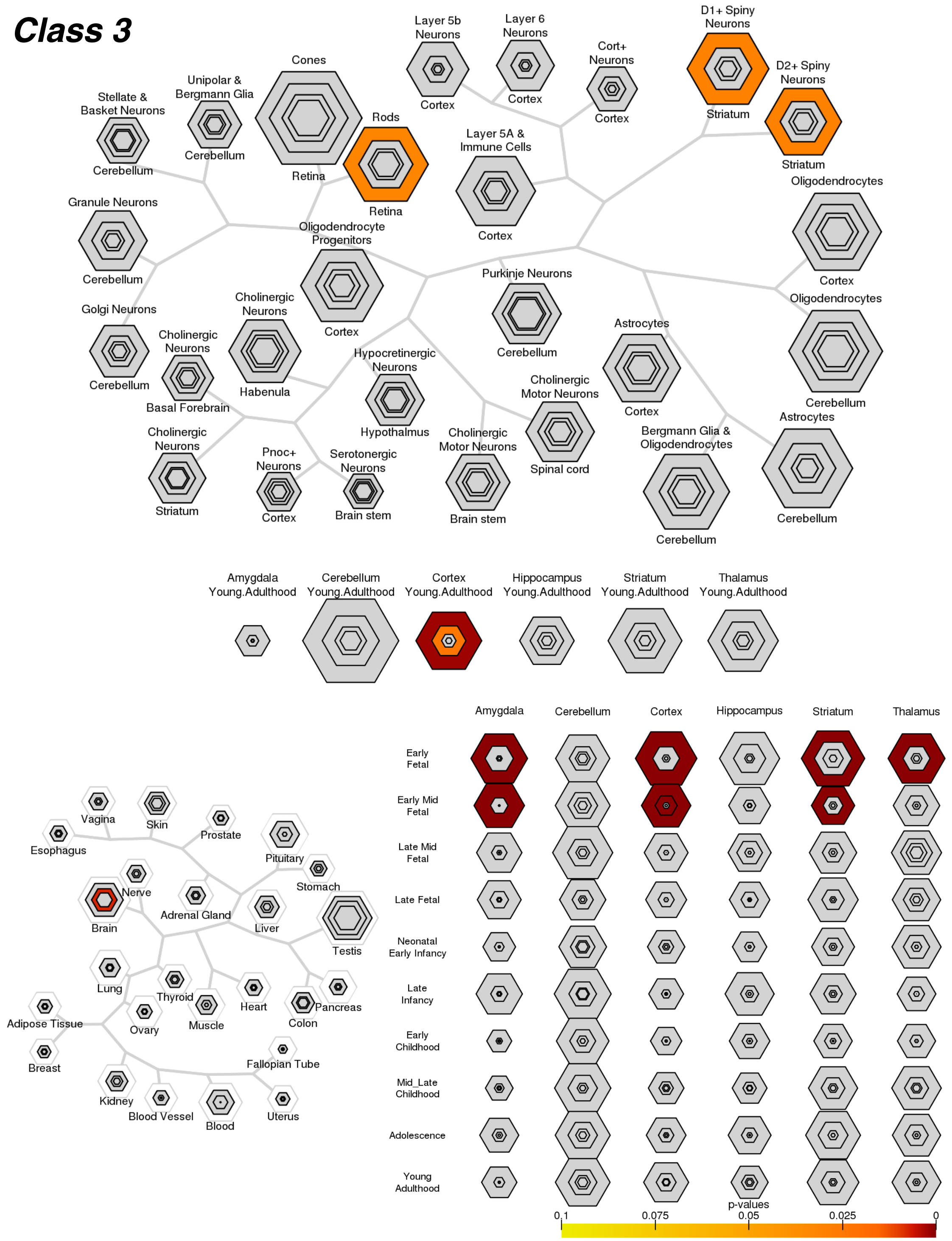

Figure S7. *Specific expression analyses profiles for Class 1, 2, and 3 modules. Significance of overlap of provided gene lists with transcripts enriched in specific cell-types or tissue types are indicated by intensity of color.*

Supplementary Information

Table S1. Neurodevelopmental phenotypes associated with seed genes. Autism (ASD), intellectual disability (ID), and developmental disability (DD) associations are listed according to the SFARI Gene database (gene score of 1 or 2), Online Mendelian Inheritance in Man (OMIM), Developmental Disorders Genotype-Phenotype Database (DDG2P), and literature. Epilepsy phenotypes are retrieved from OMIM, the DDG2P, and literature. Number of genes in modules associated with autism, ID, or DD (G_D), or epilepsy (G_E) and total number of genes in the module (G_T) including seed gene are shown.

	ASD, ID, DD	G_D	Epilepsy	G_E	G_T
Strong epilepsy association: *Class 1*					
ARHGEF9		12	(3-5), OMIM	10	44
ALDH7A1		3	(3-5), OMIM	3	48
ALG13		3	(3-5), OMIM	3	53
CACNA1A			(3-5), OMIM		
CACNA1H	(1)	16	(3-5), OMIM	5	69
CACNB4		12	(3-5), OMIM	13	41
CDKL5	(2)	14	(3-5), OMIM	8	36
CHD2	(1, 2, 9, 30)	25	(3-5), OMIM	7	72
CHRNA2			(3-5), OMIM		
CHRNA4			(3-5), OMIM		
CHRNB2		6	(3-5), OMIM	8	43
DEPDC5		8	(3-5), OMIM	4	50
DNM1	(2)	8	(3-5), OMIM	9	44
EEF1A2	OMIM, (2)	3	(3-5), OMIM	4	32

GABRA1		10	(3-5), OMIM	15	36
GABRB3	(1, 2, 14, 30)	11	(3-5), OMIM	7	63
GABRG2		9	(3-5), OMIM	15	44
GNAO1	OMIM, (2)	10	(3-5), OMIM	10	41
GRIN2A	OMIM	14	(3-5), OMIM	11	40
GRIN2B	OMIM, (1, 2, 5, 9, 14, 30)	13	(3-5), OMIM	10	37
HCN1		13	(3-5), OMIM	17	42
KCNB1		12	(3-5), OMIM	10	39
KCNMA1	OMIM	16	(3-5), OMIM	15	39
KCNQ2	(2)	8	(3-5), OMIM	6	39
KCNT1		7	(3-5), OMIM	12	37
KCTD7		12	(3-5), OMIM	6	67
LGI1		8	(3-5), OMIM	10	41
PCDH19		17	(3-5), OMIM	11	62
PRRT2	(5)	5	(3-5), OMIM	6	40
SCN1A	(2)	12	(3-5), OMIM	14	36
SCN1B		3	(3-5), OMIM	10	26
SCN2A	(1, 2, 5, 14, 30)	15	(3-5), OMIM	10	42
SCN8A	(5)	14	(3-5), OMIM	17	41
SLC1A2			(3-5), OMIM		
SLC25A22		6	(3-5), OMIM	9	42
SPTAN1		17	(3-5), OMIM	7	60
STX1B		7	(3-5), OMIM	12	38
STXBP1	(2)	7	(3-5), OMIM	10	40
SZT2			(3-5), OMIM		
TBC1D24		10	(3-5), OMIM	8	48
WWOX			(3-5), OMIM		

Moderate epilepsy association: *Class 2*					
ASH1L	OMIM, (1, 5, 30)	12	(3)	7	45
BCKDK	(1)	1	(3)	2	37
CACNA1D	(1)	13	(5), OMIM	14	40
CNTNAP2	OMIM, (1)	8	(3, 5), OMIM	7	49
DIP2C	(1)	18	(3)	9	73
DYRK1A	OMIM, (1, 2, 5, 14, 30)	12	(3)	5	56
FLNA	OMIM	8	(3, 5)	3	69
FMR1	OMIM, (5)	7	(5), OMIM	4	50
GRIN1	OMIM	8	(3, 5)	13	45
HNRNPU	(2)	12	(3, 5), OMIM	3	65
KMT2A	(1, 2, 5)	20	(3)	7	66
MBOAT7	OMIM, (1, 5)	7	(5)	10	47
MECP2	(1, 2, 5)	17	(3)	8	80
NECAP1		8	(3, 4), OMIM	7	50
NEDD4L		15	(3)	5	77
PTEN	OMIM, (1, 2, 5, 9, 14, 30)	15	(3)	5	69
RANBP17	(1, 30)	3	(3)	4	53
RELN	(1, 14)		OMIM		
SCN9A	(1)	7	(3, 4), OMIM	5	36
SLC6A1	(1, 2, 30)	7	(4, 5), OMIM	10	36
SYNGAP1	OMIM, (1, 2, 5, 9, 14, 30)	8	(3, 5)	5	37
TRIO	OMIM, (1, 5)	13	(3)	7	70
Weak epilepsy association: *Class 3*					
ADNP	OMIM, (1, 2, 5, 9, 14, 30)	17		5	73
ANK2	(1, 14, 30)	12		8	40
ANKRD11	OMIM, (1, 2, 5)	9		5	39

ARID1B	OMIM, (1, 2, 5, 14, 30)	26		7	70
ASXL3	OMIM, (1, 2, 5, 14)	12		8	67
BAZ2B	(1)	18		7	72
BCL11A	OMIM, (1, 2, 5, 14, 30)	8		4	59
CACNA2D3	(1, 14)				
CHD8	OMIM, (1, 2, 5, 9, 14, 30)	23		9	73
CIC	OMIM, (1)	11		8	36
CNTN4	(1)				
CTNND2	(1)	8		5	50
CUL3	(1, 5, 14, 30)	10		5	63
DDX3X	OMIM, (1, 2, 5)	17		5	70
DEAF1	OMIM, (1, 5)				
DSCAM	(1, 30)	8		4	47
ERBIN	(1)	6		3	54
FOXP1	OMIM, (1, 2, 5, 30)				
GIGYF2	(1, 30)	18		7	61
GRIA1	(1)	10		7	63
GRIP1	(1)	11		4	70
ILF2	(1, 30)	12		3	65
INTS6	(1)	11		3	79
IRF2BPL	(1, 30)	15		6	76
KAT2B	(1, 30)				
KATNAL2	(1, 14, 30)				
KDM5B	(1, 2, 5, 30)	15		3	76
KDM6A	OMIM, (1, 5)	19		3	72
KMT2C	OMIM, (1, 5, 30)	20		6	66
KMT5B	(1, 5)	15		4	76

LEO1	(1)	5		3	79
MAGEL2	(1)				
MED13	(1)	21		7	80
MED13L	OMIM, (1, 2, 5)	15		5	62
MET	(1)	12		11	40
MSNP1AS	(1)				
MYT1L	OMIM, (1, 2, 5, 30)	14		9	61
NAA15	OMIM, (1)	12		4	74
NCKAP1	(1, 30)	6		4	548
NLGN3	OMIM, (1, 5)	8		4	54
NRXN1	OMIM, (1, 5, 30)	16		9	58
PHF3	(1)	18		7	73
POGZ	OMIM, (1, 2, 5, 14, 30)	25		6	70
PTCHD1	OMIM, (1, 5)				
RIMS1	(1)	13		12	36
SETD5	OMIM, (1, 2, 5, 30)	21		7	63
SHANK2	OMIM, (1, 5, 30)	10		7	40
SHANK3	OMIM, (1, 5, 14, 30)	9		11	39
SMARCC2	(1)	17		6	75
SPAST	(1, 30)	15		4	75
SRCAP	OMIM, (1, 5)	18		8	60
SRSF11	(1)	4		4	64
TAOK2	(1)	5		3	40
TBL1XR1	OMIM, (1, 2, 5)	14		6	68
TBR1	(1, 5, 9, 14, 30)	11		3	53
TCF20	(1, 2)	23		9	80
TNRC6B	(1, 30)	12		6	71

TRIP12	OMIM, (1, 5, 9, 30)	16		3	76
UBN2	(1)	17		7	58
UPF3B	OMIM, (1, 5)	4		3	59
USP15	(1)	8		4	66
USP7	(1, 5)	14		4	65
WAC	(1, 2, 5, 30)	5		4	40
WDFY3	(1, 30)	11		6	57

References

1. Abrahams BS, Arking DE, Campbell DB, Mefford HC, Morrow EM, Weiss LA, et al. SFARI Gene 2.0: a community-driven knowledgebase for the autism spectrum disorders (ASDs). Mol Autism. 2013;4.
2. Deciphering Developmental Disorders S. Prevalence and architecture of de novo mutations in developmental disorders. Nature. 2017;542(7642):433-8.
3. Ran X, Li J, Shao Q, Chen H, Lin Z, Sun ZS, et al. EpilepsyGene: a genetic resource for genes and mutations related to epilepsy. Nucleic Acids Res. 2015;43(Database issue):D893-9.
4. Wang J, Lin ZJ, Liu L, Xu HQ, Shi YW, Yi YH, et al. Epilepsy-associated genes. Seizure. 2017;44:11-20.
5. Wright CF, Fitzgerald TW, Jones WD, Clayton S, Mcrae JF, van Kogelenberg M, et al. Genetic diagnosis of developmental disorders in the DDD study: a scalable analysis of genome-wide research data. Lancet. 2015;385(9975):1305-14.
6. Turner TN, Yi Q, Krumm N, Huddleston J, Hoekzema K, Stessman HAF, et al. NAR Breakthrough Article denovo-db: a compendium of human de novo variants. Nucleic Acids Res. 2017;45(D1):D804-D11.
7. Iossifov I, O'Roak BJ, Sanders SJ, Ronemus M, Krumm N, Levy D, et al. The contribution of de novo coding mutations to autism spectrum disorder. Nature. 2014;515(7526):216-U136.
8. Krumm N, Turner TN, Baker C, Vives L, Mohajeri K, Witherspoon K, et al. Excess of rare, inherited truncating mutations in autism. Nat Genet. 2015;47(6):582-8.
9. O'Roak BJ, Stessman HA, Boyle EA, Witherspoon KT, Martin B, Lee C, et al. Recurrent de novo mutations implicate novel genes underlying simplex autism risk. Nat Commun. 2014;5.
10. O'Roak BJ, Vives L, Fu W, Egertson JD, Stanaway IB, Phelps IG, et al. Multiplex targeted sequencing identifies recurrently mutated genes in autism spectrum disorders. Science. 2012;338(6114):1619-22.
11. Turner TN, Coe BP, Dickel DE, Hoekzema K, Nelson BJ, Zody MC, et al. Genomic Patterns of De Novo Mutation in Simplex Autism. Cell. 2017;171(3):710-22 e12.
12. Turner TN, Hormozdiari F, Duyzend MH, McClymont SA, Hook PW, Iossifov I, et al. Genome Sequencing of Autism-Affected Families Reveals Disruption of Putative Noncoding Regulatory DNA. Am J Hum Genet. 2016;98(1):58-74.
13. Werling DM, Brand H, An JY, Stone MR, Zhu L, Glessner JT, et al. An analytical framework for whole-genome sequence association studies and its implications for autism spectrum disorder. Nat Genet. 2018;50(5):727-36.
14. De Rubeis S, He X, Goldberg AP, Poultney CS, Samocha K, Cicek AE, et al. Synaptic, transcriptional and chromatin genes disrupted in autism. Nature. 2014;515(7526):209-U119.

15. Yuen RK, Merico D, Cao H, Pellecchia G, Alipanahi B, Thiruvahindrapuram B, et al. Genome-wide characteristics of de novo mutations in autism. NPJ Genom Med. 2016;1:160271-1602710.
16. Yuen RKC, Merico D, Bookman M, Howe JL, Thiruvahindrapuram B, Patel RV, et al. Whole genome sequencing resource identifies 18 new candidate genes for autism spectrum disorder. Nat Neurosci. 2017;20(4):602-+.
17. Epi KC, Epilepsy Phenome/Genome P, Allen AS, Berkovic SF, Cossette P, Delanty N, et al. De novo mutations in epileptic encephalopathies. Nature. 2013;501(7466):217-21.
18. de Ligt J, Willemsen MH, van Bon BW, Kleefstra T, Yntema HG, Kroes T, et al. Diagnostic exome sequencing in persons with severe intellectual disability. N Engl J Med. 2012;367(20):1921-9.
19. Halvardson J, Zhao JJ, Zaghlool A, Wentzel C, Georgii-Hemming P, Mansson E, et al. Mutations in HECW2 are associated with intellectual disability and epilepsy. J Med Genet. 2016;53(10):697-704.
20. Rauch A, Wieczorek D, Graf E, Wieland T, Endele S, Schwarzmayr T, et al. Range of genetic mutations associated with severe non-syndromic sporadic intellectual disability: an exome sequencing study. Lancet. 2012;380(9854):1674-82.
21. Lelieveld SH, Reijnders MR, Pfundt R, Yntema HG, Kamsteeg EJ, de Vries P, et al. Meta-analysis of 2,104 trios provides support for 10 new genes for intellectual disability. Nat Neurosci. 2016;19(9):1194-6.
22. Fromer M, Pocklington AJ, Kavanagh DH, Williams HJ, Dwyer S, Gormley P, et al. De novo mutations in schizophrenia implicate synaptic networks. Nature. 2014;506(7487):179-84.
23. Gulsuner S, Walsh T, Watts AC, Lee MK, Thornton AM, Casadei S, et al. Spatial and temporal mapping of de novo mutations in schizophrenia to a fetal prefrontal cortical network. Cell. 2013;154(3):518-29.
24. Kranz TM, Harroch S, Manor O, Lichtenberg P, Friedlander Y, Seandel M, et al. De novo mutations from sporadic schizophrenia cases highlight important signaling genes in an independent sample. Schizophr Res. 2015;166(1-3):119-24.
25. McCarthy SE, Gillis J, Kramer M, Lihm J, Yoon S, Berstein Y, et al. De novo mutations in schizophrenia implicate chromatin remodeling and support a genetic overlap with autism and intellectual disability. Mol Psychiatr. 2014;19(6):652-8.
26. Smedemark-Margulies N, Brownstein CA, Vargas S, Tembulkar SK, Towne MC, Shi J, et al. A novel de novo mutation in ATP1A3 and childhood-onset schizophrenia. Cold Spring Harb Mol Case Stud. 2016;2(5):a001008.
27. Fischbach GD, Lord C. The Simons Simplex Collection: A Resource for Identification of Autism Genetic Risk Factors. Neuron. 2010;68(2):192-5.
28. Collaborative E, Abou-Khalil B, Alldredge B, Bautista J, Berkovic S, Bluvstein J, et al. The epilepsy phenome/genome project. Clin Trials. 2013;10(4):568-86.
29. Mcrae JF, Clayton S, Fitzgerald TW, Kaplanis J, Prigmore E, Rajan D, et al. Prevalence and architecture of de novo mutations in developmental disorders. Nature. 2017;542(7642):433-+.
30. Sanders SJ, Xin H, Willsey AJ, Ercan-Sencicek AG, Samocha KE, Cicek AE, et al. Insights into Autism Spectrum Disorder Genomic Architecture and Biology from 71 Risk Loci. Neuron. 2015;87(6):1215-33.
31. Coe BP, Witherspoon K, Rosenfeld JA, van Bon BWM, Vulto-van Silfhout AT, Bosco P, et al. Refining analyses of copy number variation identifies specific genes associated with developmental delay. Nat Genet. 2014;46(10):1063-71.
32. Fabregat A, Sidiropoulos K, Garapati P, Gillespie M, Hausmann K, Haw R, et al. The Reactome pathway Knowledgebase. Nucleic Acids Res. 2016;44(D1):D481-D7.
33. Kuleshov MV, Jones MR, Rouillard AD, Fernandez NF, Duan Q, Wang Z, et al. Enrichr: a comprehensive gene set enrichment analysis web server 2016 update. Nucleic Acids Res. 2016;44(W1):W90-7.
34. Xu XX, Wells AB, O'Brien DR, Nehorai A, Dougherty JD. Cell Type-Specific Expression Analysis to Identify Putative Cellular Mechanisms for Neurogenetic Disorders. J Neurosci. 2014;34(4):1420-31.
